# CellECT: cell evolution capturing tool

**DOI:** 10.1186/s12859-016-0927-7

**Published:** 2016-02-17

**Authors:** Diana L. Delibaltov, Utkarsh Gaur, Jennifer Kim, Matthew Kourakis, Erin Newman-Smith, William Smith, Samuel A. Belteton, Daniel B. Szymanski, B. S. Manjunath

**Affiliations:** Department of Electrical and Computer Engineering, University of California, Santa Barbara, Santa Barbara, CA USA; Department of Molecular, Cellular, and Developmental Biology, University of California, Santa Barbara, Santa Barbara, CA USA; Department of Botany and Plant Pathology, Purdue University, West Lafayette, IN USA; Biological Sciences, Purdue University, West Lafayette, IN USA

**Keywords:** Interactive segmentation, 3D microscopy, Analysis

## Abstract

**Background:**

Robust methods for the segmentation and analysis of cells in 3D time sequences (3D+t) are critical for quantitative cell biology. While many automated methods for segmentation perform very well, few generalize reliably to diverse datasets. Such automated methods could significantly benefit from at least minimal user guidance. Identification and correction of segmentation errors in time-series data is of prime importance for proper validation of the subsequent analysis. The primary contribution of this work is a novel method for interactive segmentation and analysis of microscopy data, which learns from and guides user interactions to improve overall segmentation.

**Results:**

We introduce an interactive cell analysis application, called *CellECT*, for 3D+t microscopy datasets. The core segmentation tool is watershed-based and allows the user to add, remove or modify existing segments by means of manipulating guidance markers. A confidence metric learns from the user interaction and highlights regions of uncertainty in the segmentation for the user’s attention. User corrected segmentations are then propagated to neighboring time points. The analysis tool computes local and global statistics for various cell measurements over the time sequence. Detailed results on two large datasets containing membrane and nuclei data are presented: a 3D+t confocal microscopy dataset of the ascidian *Phallusia mammillata* consisting of 18 time points, and a 3D+t single plane illumination microscopy (SPIM) dataset consisting of 192 time points. Additionally, *CellECT* was used to segment a large population of jigsaw-puzzle shaped epidermal cells from *Arabidopsis thaliana* leaves. The cell coordinates obtained using *CellECT* are compared to those of manually segmented cells.

**Conclusions:**

*CellECT* provides tools for convenient segmentation and analysis of 3D+t membrane datasets by incorporating human interaction into automated algorithms. Users can modify segmentation results through the help of guidance markers, and an adaptive confidence metric highlights problematic regions. Segmentations can be propagated to multiple time points, and once a segmentation is available for a time sequence cells can be analyzed to observe trends. The segmentation and analysis tools presented here generalize well to membrane or cell wall volumetric time series datasets.

**Electronic supplementary material:**

The online version of this article (doi:10.1186/s12859-016-0927-7) contains supplementary material, which is available to authorized users.

## Background

**Fig. 1:**
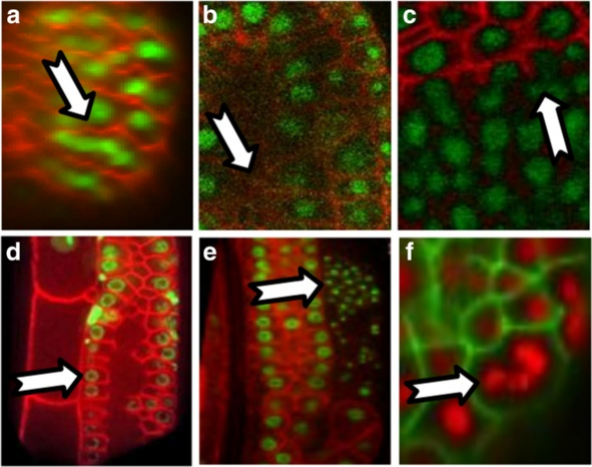
Image segmentation challenges. **a** Reconstructed cross-section of a SPIM light sheet microscopy volume of the ascidian *P. mammillata* showing an artifact in which as many as five nuclei appear connected. This makes it difficult for existing nuclei detection methods to properly segment. **b** Weak signal in the membrane channel in lower *z* slices of a confocal microscopy image. **c** Inconsistent signal strength in the cell wall channel of a *z* slice through a confocal microscopy image of *Arabidopsis thaliana* (image courtesy Elliot Meyerowitz Lab, Division of Biology, California Institute of Technology). **d** Cells with interrupted membrane which share cytoplasm, as in this example of the *Caenorhabditis elegans* gonad cells [32]. Watershed segmentation methods will have difficulty segmenting such structures due to leakage. **e** Sperm cells appear in the nuclei channel resulting in false positives for a nuclei detector [32]. **f** Dividing *P. mammillata* cell SPIM images that show up as large nuclei

Fluorescent microscopy datasets composed of nuclear and membrane (or cell wall) channels pose problems to automated image analysis algorithms, as seen in the examples in Fig. 1. To address these challenges we propose an interactive segmentation and analysis tool that guides the user to quickly correct potential errors and adaptively propagate these corrections through the entire dataset, thus providing a robust framework for further quantitative image analysis and results validation.

Interactive segmentation has gained significant interest in the bio-imaging community in recent years. For example, [1] proposes an interactive learning approach for segmentation of histological images. *Ilastik* is a widely used interactive segmentation and classification tool [2]. Other tools are specifically targeted to, for example electron microscopy images [3] or for segmentation of clusters of cells such as [4] which classifies pixels based on the geodesic commute distance and spectral graph theory. The user-guided segmentation algorithm in [5] is aimed at 3D nuclei segmentation and integrates multiple nuclei models simultaneously. The software introduced in [6] offers interactive visualization and analysis tools which enable users to create a processing pipeline for microscopy applications, from image filtering to segmentation and analysis. The work of [7] uses an active contour approach based on parametrized B-splines for interactive 3D segmentation. A conditional random field whose underlying graph is a watershed merging tree is trained in the interactive segmentation approach of [8] and is applied to segmentation of neuronal structures in electron microscopy data.

**Fig. 2:**
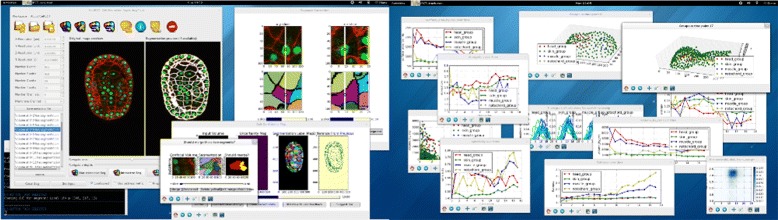
CellECT software screenshots. *CellECT* enables the interactive segmentation and analysis of 3D+t microscopy membrane (or cell wall) volumes. Screenshots of *CellECT*’s main interface (*left-most*), the interactive segmentation tool (*left-middle*), and analysis module (*right*) are shown above

Here we introduce an interactive cell analysis application called *CellECT* (Fig. 2), which consists of a segmentation component and an analysis component. The user can modify a label map that is obtained using seeded Watershed [9], by adding, removing or modifying segments. The algorithm aims at obtaining correct segmentation with minimum user interaction. We define an adaptive metric we call *cellness* which is trained to highlight the regions where the segmentation is likely to be incorrect and may require the user’s attention. Additionally, the algorithm can offer specific suggestions. Segmentation results can then be propagated to other time points in the 3D+t dataset. Furthermore, *CellECT* provides an analysis component which summarizes the changes in various cell measurements over the time sequence. A user-friendly interface allows for easy workspace management, including the import of 3D or 3D+t TIFF stacks with any additional information (e.g. metadata such as scale, nuclei detection, or anterior-posterior axis of the specimen), opening an existing workspace for continuing work, or appending two existing workspaces to concatenate time points from separate TIFF files.

The primary contributions include: (1) an interactive segmentation tool that manages user guidance markers in the geodesic image space, (2) an adaptive *cellness* metric that learns from user-feedback and computes/maintains a probabilistic belief about the quality of a cell’s segmentation and a method to make suggestions to the user, (3) the ability to propagate user corrections to other time points, and (4) an analysis component which facilitates quantitative observation about the organism’s development changes over a time sequence. These algorithms and features are packaged into an open source software application. We utilize this software for the analysis of a 3D+t confocal microscopy dataset of the ascidian *P. mammillata* consisting of 18 time point, a 3D+t SPIM dataset of *P. mammillata* consisting of 192 time points, and a dataset of eight 2D confocal microscopy slices of *A. thaliana* consisting of 112 pavement cells.

## Methods

### *CellECT* overview

*CellECT* is an application for interactive segmentation and analysis of 3D+t microscopy datasets containing cell boundary information (e.g., plasma membrane or cell wall). Its features include:

**Workspace management:***CellECT* allows users to import a dataset in TIFF format along with other optional information such as image metadata, seed points (e.g. nuclei) obtained with an external tool (e.g. a nuclei detector application), or sample orientation (e.g., anterior-posterior axis). *CellECT* then creates a workspace consisting of individual time points and channels.

Users may choose to append existing workspaces to the current one in order to concatenate additional time points. This facilitates the construction of time sequences from otherwise independent image stacks, and is especially useful when working with large time sequences.

**Input seeds:** Input seeds for the segmentation algorithm may be loaded if available, or they can be added by the user when working on the segmentation. Furthermore, if the dataset represents a time sequence, such input seeds can be propagated from neighboring time points.

**Interactive segmentation:** Interactions include modifying, deleting, and adding segments. We introduce a *cellness* confidence measure that models the segmented cells’ features and reas of uncertainty for the user’s attention. Additionally, the interactive segmentation tool suggests corrections for the user to accept or reject.

**High-throughput analysis:***CellECT* provides the option of running the segmentation tool in a non-interactive mode on multiple time points. This option is useful for high-throughput analyses where results are needed quickly with little human intervention. Further, an option to propagate the segmentation to neighboring time points is available. This transfers seed points from one time point to the next as well as propagates a bias for segment shapes. The user has the option to correct the segmentation results in the interactive mode.

**Analysis tool:***CellECT* provides an analysis module which runs on a selected subset of time points for which segmentation is available. Changes in local and global statistics of various cell measurements over time are tracked. The user has the ability to select regions of interest in order to observe cell behavior. Furthermore, automated clustering can be performed to categorize similar cells.

**Exporting results:** The segmentation results are available in various formats: slice by slice TIFF files, MAT-file format, and polygon contours in XML files. The analysis and measurements computed for each segmented cell are available in XML format.

### Interactive segmentation

The method starts out with the membrane/cell wall channel of the original microscopy volume, *V*_*t*_, at a given time point *t*, and a point cloud of initialization seeds (nuclei) associated with this volume, . These points, if available, may be imported from an external nuclei detector such as described by [10], or randomly distributed throughout the volume, or propagated from the segmentation available at a neighboring time point.

In this section, we limit the discussion to the interactive segmentation, analysis and confidence evaluation of a single volume at a given time *t* and use the simplified notation of *V* and  for *V*_*t*_ and  respectively. Though this is not a limitation on the overall methodology, the notation in the analysis that follows refers to a single time point *t*.

At each iteration the user contributes a set of guidance marker points, $\mathcal {P}^{i}$, where each marker point **x**_*p*_ is described by its spatial coordinates within the given volume: **x**_*p*_=[*x,y*,*z*]. The initial input seed points, , along with guidance marker points resulting from subsequent user interaction are maintained in a graph $G^{i} = \left \{\mathcal {V}^{i}, \mathcal {E}^{i}\right \}$, where *i* is the user interaction iteration index, and . Each seed $\mathbf {x}_{p} \in \mathcal {V}^{i}$ is associated with a segment in the final segmentation, and each segment is described by at least one seed. The index of the segment associated with a seed is given by Seg(**x**_*p*_)=*k*. There exists an edge in the graph $e_{\textit {pq}} \in \mathcal {E}^{i}$ if Seg(**x**_*p*_)=Seg(**x**_*q*_). In summary, the nodes in the graph represent seed points, and the edges in the graph model the membership of seeds to disjoint subsets (connected components in the graph). Each disjoint subset contains the seeds (nuclei and user guidance markers) associated with one segment.

User interactions model such actions as “merge two segments”, “modify a segment”, “delete a segment”, and “add a new segment”, by manipulating the graph of input markers *G*^*i*^. For example, to modify a segment additional guidance markers (seeds) can be provided and associated with the given segment. To delete a segment, the seeds associated with it are eliminated. The seeds (nuclei and user markers) and their subset membership are efficiently maintained as a disjoint set data structure [11], permitting find and union operations with computational complexity $O(log \mathcal {V}^{i})$ and constant amortized complexity. The graph which is actually is actually a forest of tree graphs, is implemented internally using two arrays.

At each iteration *i*, a seeded Watershed segmentation method [12] takes as input a membrane (or cell wall) volume *V* and a spatial arrangement of seed markers along with their subset membership relationship as modeled by the configuration of graph *G*^*i*^. This results in a segmentation label map, *S*^*i*^, in which every pixel is assigned a label corresponding to a segmented cell or the image background.

The segmentation label map *S*^*i*^ is evaluated by computing the *cellness* metric of each segment (detailed in Section “Learning a cellness metric”). The *cellness* metric uses segment measurements to return a confidence value which models the likelihood that the segment correctly represents a segmented cell. The *cellness* metric is used to highlight regions of uncertainty for the user’s attention.

Once the segmentation label map and the *cellness* metric evaluation of each segment are computed, the user may once again modify the segmentation. The process repeats until the user is satisfied with the results.

#### Watershed markers

User guidance seeds play a vital role in initializing the seeded watershed segmentation algorithm. The watershed algorithm is a segmentation algorithm which flood fills the image space starting from input markers, interpreting the image as a topological relief, where pixel intensity is analogous to altitude. The input markers may be a single point per segment or a series of strokes through the segment volume. *CellECT* enables users to interact with the watershed segmentation algorithm by manipulating the input markers through guidance seeds.

Each input seed point, whether detected using a nuclei detector or manually marked, translates to one input marker for the segmentation algorithm, which flood fills its neighborhood to form a segment. In order to modify a segment, the user places additional guidance seeds at each iteration *i*, which are maintained in the graph *G*^*i*^.

When multiple guidance seeds are provided for the same segment, *CellECT* combines them to form stroke markers in the 3D space. This is done by computing the geodesic shortest path in the image space between one central seed and all other seeds describing the same segment *k*. The central seed, given by image space coordinates $\mathbf {x}_{k}^{c}$, is picked to minimize the Euclidean distance to the mean location $\overline {\mathbf {x}}$ of all the seeds $\mathbf {x} \in \mathcal {V}^{i}_{k}$ describing a segment *k*:

(1) $$ \mathbf{x}_{k}^{c} = \underset{\mathbf{x} \in \mathcal{V}^{i}_{k}}{\arg\!\min} || \mathbf{x} - \overline{\mathbf{x}} ||  $$

The geodesic shortest path between the seed $\mathbf {x}_{k}^{c}$ and the other seeds pertaining to segment *k*, $\mathcal {V}^{i}_{k} \setminus \left \{\mathbf {x}_{k}^{c}\right \}$, connects all seeds describing to segment *k*. The shortest path is computed using gradient descent over the distance function $\mathcal {D}$. $\mathcal {D}$ can be obtained by solving the Eikonal Eq. 2 using the *Fast Marching* algorithm introduced in [13].

(2) $$ |\text{grad} (\mathcal{D}) |=P  $$

where *P*(·) is the speed of the propagating front, embedded in a higher dimensional level set function. If the speed *P* is constant, the resulting distance function $\mathcal {D}$ can be seen as the distance function to the starting point, $\mathbf {x}_{k}^{c}$. Gradient descent on this distance function returns the shortest path in geodesic image space from each point in $\mathcal {V}^{i}_{k} \setminus \{\mathbf {x}_{k}^{c}\}$ to the starting point $\mathbf {x}_{k}^{c}$.

**Fig. 3:**
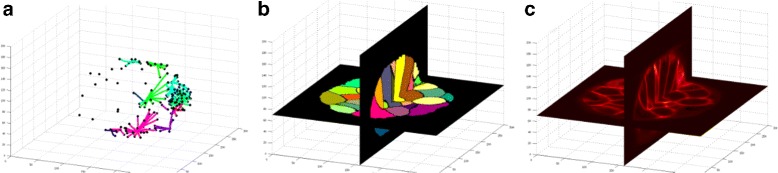
User markers. **a** Seeds (in black), and the shortest path through the geodesic image space between identically-labelled seeds. These seeds and paths are given as initialization points or strokes to a seeded Watershed algorithm. **b** An *x*−*y* and an *x*−*z* plane through the resulting segmentation. **c** the corresponding planes through the original image

The resulting shortest paths connecting the guidance markers for each segment together with the point cloud of input seeds are given as input markers to the Watershed algorithm. Figure 3 shows an example of such input guidance markers, the resulting segmentation and the original image. Guidance markers pertaining to the same cell must be connected to each other in order to prevent the segmentation of one to result in disjoint fragments. The geodesic shortest path connects seeds through curved paths without crossing cell boundaries.

#### Segment features

Once the segmentation is computed using the input markers described in Section “Watershed markers”, several properties are calculated for each segment at every iteration. These features are further used in calculating the *cellness* metric (Section “Learning a cellness metric”) to guide user interactions and for computing cell statistics (Section “*CellECT* recommendations, segment propagation and cell analysis”).

##### Segment border features

capture the implicit assumption that the membrane or cell wall channels are expected to have higher intensity signal than the cell interiors.

*1) Border to interior intensity ratio* is given by $\frac {\overline {\mathcal {B}_{k}}} {\overline {\mathcal {S}_{k}}}$, where $\overline {\mathcal {B}_{k}}$ represents the average intensity along the border of segment *k* and $\overline {\mathcal {S}_{k}}$ represents the average signal intensity within segment *k*.

*2) Distance between border and interior intensity histograms.* This is computed using the Earth Mover’s Distance [14] and evaluates whether there is any membrane signal present in the segment interior.

##### Position properties

describe the segment’s position in the image space and relative to the specimen’s coordinate system, as explained below.

*3) Segment centroid.* The centroid coordinates are given with respect to the image origin, rescaled according to the image resolution in each dimension, and is equivalent to the center of mass of the segment.

*4) Centroid distance from specimen surface.* The minimum Euclidean distance of each segment’s centroid to the specimen’s outer boundary, scaled by the image resolution, is computed. This is given by $ D^{\mathcal {B}}_{\mathcal {B}_{k}}$, where $\mathcal {B}$ is the boundary (surface) of the specimen in the microscopy volume, $\mathcal {B}_{k}$ is the boundary of segment *k*, and the distance transform in Eq. 3 denotes the minimum distance of every point **x** in set *A* to the points in set *B*.

(3) $$ {D_{A}^{B}} = \left\{\underset{\mathbf{p} \in B}{\min}\, d(\mathbf{x},\mathbf{p})\ | \mathbf{x} \in A\right\}   $$

*5) Best fit line.* A line is fit through the voxel coordinates of segment *k* using the algorithm of [15], which is based on the M-estimator technique that iteratively fits the line using the weighted least-squares algorithm. The resulting feature consists of a 6-element vector containing a normalized unit vector collinear to the line and a point on the line, in the image coordinate system.

*6) Segment position along the anterior-posterior (AP) axis.* The AP axis is a set of points $\mathcal {S}_{\textit {AP}}$ obtained by interpolating a list of consecutive marker points in the image space given by the user, which traverses the specimen from the anterior end to the posterior end. The projection of the centroid of segment *k*, *c*_*k*_ is given by the point on the AP axis with the smallest Euclidean distance to *c*_*k*_. This is given by Eq. 4. Next, the position along the AP axis is calculated as the percentage along the axis starting from the first anterior point to the last posterior point.

(4) $$ \mathbf{c}_{k}^{AP} = \underset{\mathbf{x} \in \mathcal{S}_{AP}}{\arg\!\min} ||\mathbf{x} - \mathbf{c}_{k}||   $$

*7) Segment angle with the AP axis.* For every segment *k*, the unit tangent to the AP axis at the projection point $\mathbf {c}_{k}^{AP}$ is used to compute the smallest angle with the best fit line unit vector.

*8) Segment distance to AP axis.* This is given by $||\mathbf {c}_{k}^{AP} - \mathbf {c}_{k}||$.

##### Shape and size properties

characterize the 3-D shape of each segment.

*9) Segment volume:* is given by the voxel count of the segment scaled by the image resolution, $V_{k} = |\mathcal {S}_{k}| \cdot \mu _{x} \mu _{y} \mu _{z}$, where $|\mathcal {S}_{k}|$ is the cardinality of the set of voxels occupied by segment *k*, and *μ*_*x*_, *μ*_*y*_ and *μ*_*z*_ represent the image resolution scale factor in each dimension.

*10) Distance of segment border to segment centroid:* This feature computes the histogram of the set of distances between the voxels on the segment border and the segment centroid, ${D_{k}^{c}} = \{ ||\mathbf {x} - \mathbf {c}_{k}|| \cdot \mu | \mathbf {x} \in \mathcal {B}_{k}\}$, where *D* is the distance function defined in Eq. 3. Here, $\mu = \frac {1}{\underset {\textbf {x} \in \mathcal {B}_{k}}{\text {max}} ||\mathbf {x} - \mathbf {c}_{k}||}$ is a scale factor such that the maximum element in the set is 1.

*11) Sphericity:* The radius of a sphere with the same volume as segment *k* is given by $r_{k} = \sqrt [3]{\frac {3 \cdot |\mathcal {B}_{k}|}{4 \cdot \pi }}$. The ratio of the surface area of this sphere to the surface area $\mathcal {B}_{k}$ of segment *k* is stored as a feature and indicates how much the segment shape deviates from a sphere, and is given by $\frac {|\mathcal {S}_{k}|}{4\pi \cdot {r_{k}^{2}}}$.

*12) Squareness:* is given by the ratio of the segment volume and the volume of the minimum enclosing bounding box: $ \frac {V_{k}}{V^{\text {box}}_{k}}$. The minimum oriented bounding box is obtained from the projection extremities of each segment along each of the three principal axes.

*13) Cylindricity:* This metric evaluates the segment’s deviation from a cylinder. The volume $V^{\text {cyl}}_{k}$ of the minimum enclosing cylinder oriented along the principal axes of segment *k*. The lowest $ \frac {V_{k}}{V^{\text {cyl}}_{k}}$ ratio of the three enclosing cylinders represents the cylindricity score.

*14) Convexity:* The deviation of the segment shape from a convex form is measured as the ratio of the segment volume to the convex hull volume: $\frac {V_{k}}{V_{k}^{\text {hull}}}$.

*15) Entropy:* is a measure of compactness and is calculated using the eigen values obtained from principal component analysis, as in [16].

*16) Elongation:* Similar to [16], the elongation is given by the ratio of the largest eigenvalue to the midium eigenvalue: $\frac {\lambda _{\text {max}}}{\lambda _{\text {med}}}$.

*17) Flatness:* Similar to [16], the elongation is given by the ratio of the medium eigenvalue to the smallest eigenvalue: $\frac {\lambda _{\text {med}}}{\lambda _{\text {min}}}$.

#### Learning a cellness metric

A novel *CellECT* feature is its ability to highlight uncertain segmentation results to the user. A confidence metric, called *cellness*, is constructed for each dataset based on a continuous learning framework that models various cell features described above. The model is continuously updated based on user interactions.

##### Expected segment characteristics

**Fig. 4:**
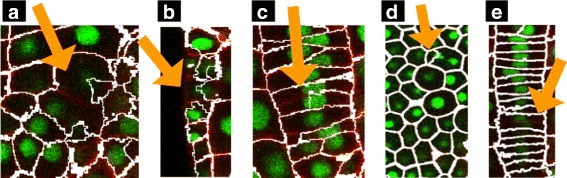
Example of problematic segments. A correct segment exhibits one or more of these characteristics: the boundary signal intensity as stronger than the interior, the common boundary with a neighboring cell has high intensity, the segment shape is almost convex and the segment’s shape is similar to that of its neighbors. Incorrect segments indicated by arrows: **a** segment is far from convex, **b** signal intensity in the membrane channel is not high on the segment border, **c** signal intensity in the membrane channel is too high in the segment interior, **d**–**e** segments are not similar to their surroundings

Figure 4 shows several examples of incorrect segments. We argue that a correctly segmented cell has at least one of these distinct characteristics: (1) the boundary of the segment has higher intensity than the interior, (2) the boundary between a cell and each of its neighboring segments has high intensity, (3) cells are mostly convex, and (4) cells are on average similar to their neighbors. Inconsistent intensity along the segment boundary is often an indicator of a segment which does not adhere to membrane or cell wall staining, for example the result of over-segmentation. The cell neighbor similarity expectation is motivated by the fact that cells usually develop in compact tissues which maintain consistent appearance. Shape and size features were chosen for cell similarity since these are the cues humans typically look for to identify incorrectly segmented cells. Viewing these measurements outside their local context and independent of each other may not be effective when working with multiple tissues, whose cells differ in appearance. Therefore, in addition to quantifying each of these properties, the *cellness* metric adapts to user feedback. Metrics (1)-(3) are obtained as explained in Section “Segment features”, and denoted ${s_{k}^{1}}$, ${s_{k}^{2}}$ and ${s_{k}^{3}}$. Metric (4), ${s_{k}^{4}}$, is computed by calculating the average distance in feature space between segment *k* and each of its neighbors *j*, as in Eq. (5). Here, *f*_*k*_ (*f*_*j*_) is the feature vector obtained by concatenating the values of *volume, sphericity, flatness,* and *elongation* obtained as described in Section “Segment features” and normalized zero mean and unit standard deviation. The scale factor *c* brings ${s_{k}^{4}}$ to range between 0 and 1. Thus, the values of ${s^{1}_{k}}$ through ${s^{4}_{k}}$ range between 0 and 1, where large values are obtained by segments which meet the characteristics (1)-(4) above.

(5) $$ {s_{k}^{4}} = 1 - \frac{\sum \limits_{j \in \mathcal{N}_{k}} c||f_{k} - f_{j}||}{|\mathcal{N}_{k}|}  $$

##### Learning region characteristics from positive user examples

User feedback is of two types: a problematic segment may be corrected, or a segment may be marked as correct. Segments marked as correct provide information about the expected segment measurements in their neighborhood. Segments in the neighborhood of positive user feedback are compared against the user examples under the spatial homogeneity assumption and scored accordingly.

**Fig. 5:**
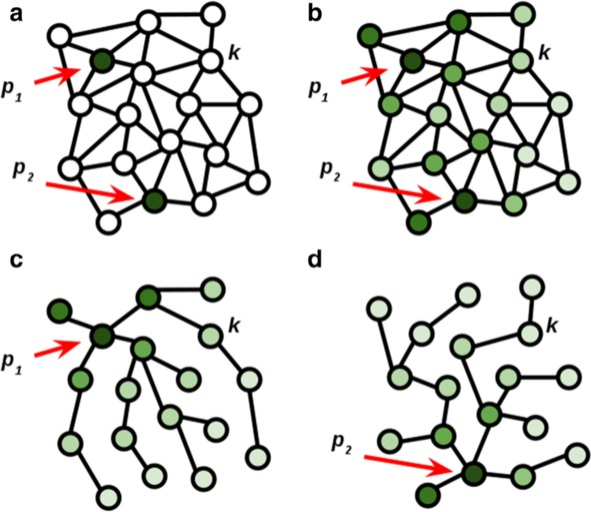
**a** Segments represented as nodes in a graph. Edges connect neighboring segment. Two segments (*p*_1_ and *p*_2_) marked by the user as correct. **b** Disseminate credit from the correct segments to other segments in the graph, based on similarity within the neighborhood. **c** Confidence credit disseminated from segment *p*_1_ to all other segments along the path of highest similarity. **d** Credit disseminated from *p*_2_

Each segment is modeled as a node in a graph and edges are introduced between nodes representing neighboring cells. Given a set of segments marked by the user as correct, the task is to disseminate this confidence credit to other segments in the graph, based on their similarity to and distance from the correct segments, as shown in Fig. 5a-b. We consider the simplified problem of disseminating the credit from each correct segment to every node in the graph along the optimal path.

The optimal path from a segment *k* to a segment *p* labeled as correct is the path which maximizes the probability that every segment along the path is correct. This can be written as in Eq. 6, in which a path through the graph between nodes (segments) *k* and *p* is defined by the set of vertexes ${P_{k}^{p}} = \left \{v_{1}, v_{2},.. v_{n} \right \}$, where *v*_1_=*p*, *v*_*n*_=*k* and *P*(*v*_1_)=1.

(6) $$ \begin{aligned} P(v_{n}|v_{1}) &= \max \limits_{{P_{k}^{p}}}P(v_{n}|v_{n-1}, v_{1}) P(v_{n-1} | v_{1}) =\\ &= \max \limits_{{P_{k}^{p}}} P(v_{n}|v_{n-1},v_{1}) P(v_{n-1}| v_{n-2},v_{1}) \\ &\qquad P(v_{n-2}|v_{n-3},v_{1})\ldots P(v_{2}|v_{1}) P(v_{1}) \end{aligned}  $$

We model the probability *P*(*v*_*i*_|*v*_*i*+1_,*v*_1_) as the pairwise similarity between the two neighboring nodes *v*_*i*_ and *v*_*i*+1_, which is independent of *v*_1_. This can be expressed as *P*(*v*_*i*_|*v*_*i*+1_,*v*_1_)=*P*(*v*_*i*_|*v*_*i*+1_)=*d*_*i,i*+1_. The similarity measure *d*_*i,i*+1_ is given by 1−||*f*_*i*_−*f*_*i*+1_||·*c*, where *f*_*i*_ and *f*_*i*+1_ are the feature vectors defined earlier and *c* is the scaling factor in Eq. (5). Therefore, the goal is to obtain the path that maximizes the pairwise similarity between segment *k* and the correctly labeled segment *p*, i.e., $\mathcal {A}\left ({P_{k}^{p}}\right) = \prod \limits _{i=1}^{n} d_{i,i+1}$.

To find the above optimal path, we note that:

(7) $$ P_{k}^{p*} = \mathop{\text{argmax}}\limits_{{P_{k}^{p}} = \left\{v_{1},.. v_{n} \right\}} \prod \limits_{i=1}^{n} d_{i,i+1} = \mathop{\text{argmin}} \limits_{{P_{k}^{p}} = \left\{v_{1},.. v_{n} \right\}} \sum \limits_{i=1}^{n} - \log d_{i,i+1}  $$

where *w*_*ij*_=− log*d*_*ij*_ are non-negative weights. Therefore, the method described by [17] can be used to obtain the shortest path from a correct node *p* to every node in the graph, as shown in Fig. 5c-d. Finally, the confidence credit obtained by a segment *k* from a set of correctly labeled segments *p* is given by:

(8) $$ {s_{k}^{5}} = \max \limits_{p} P(k|p) = \max \limits_{p} exp\left(-\mathcal{A}\left(P_{k}^{p*}\right)\right)  $$

Hence, ${s_{k}^{5}}$ quantifies the confidence that segment *k* is correct assuming the user input and the knowledge about its neighborhood, in terms of similarity metrics.

**Fig. 6:**
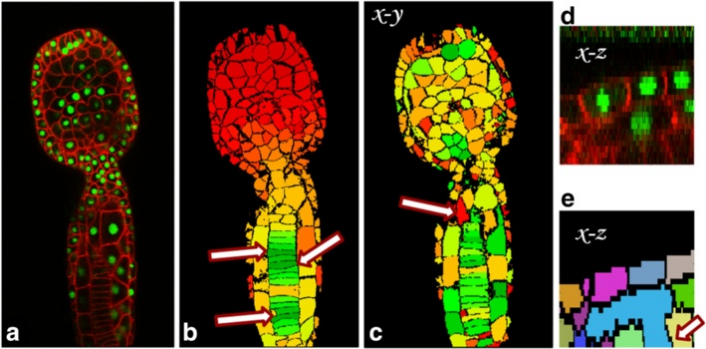
*Cellness* metric example. **a** Slice through the original confocal microscopy image. **b** Propagation of confidence from segments marked as correct (indicated by arrows) to similar neighbors. **c** Color coded *cellness* metric. **d** Reconstructed cross section in the *x*−*z* plane of the cell with low *cellness* metric indicated by arrow in panel. This segment appears correct in the view from panel C however it has a low *cellness* score. **e** Error in the segmentation, indicated by arrow, observable in the cross section (the segment leaks into the cell below). The *cellness* metric helped identify this error in segmentation

An example of the effects of this metric are shown in Fig. 6b.

##### Metric learning from positive and negative user examples

The *cellness* metric adapts to user feedback. Segments which are marked as correct are used as positive examples, while segments that are corrected through user interactions are used as negative examples. We use the semi-supervised learning approach of [18] which is designed to work well if given a very small number of labeled samples, together with a large number of unlabeled samples. The feature space consists of $\left ({s_{k}^{1}},{s_{k}^{2}},{s_{k}^{3}},{s_{k}^{4}}\right)$ tuples, and two class labels are considered: correct segment and incorrect segments. The output of the classifier is the probability that each sample belongs to the *correct* class, which we denote ${s_{k}^{6}}$.

A correct segment will obtain high values for metrics ${s_{k}^{1}}$ through ${s_{k}^{5}}$ and, if the classifier is trained well, the segment will obtain a high value for ${s^{6}_{k}}$. The *cellness* metric combines ${s_{k}^{1}}$ through ${s_{k}^{6}}$, giving more weight to either the mean of ${s_{k}^{1}}$ through ${s_{k}^{5}}$ or to the result of ${s_{k}^{6}}$, according to Eq. 9. The average of ${s_{k}^{1}}$ through ${s_{k}^{5}}$ gives equal weight to each of the five sub-metrics. The output of the classifier produces a more complex decision boundary tailored to the dataset. This score is reliable if a balanced number of positive and negative training samples are available, and sufficient training examples are available. Thus, the weight factor *ν* ranges between 0 and 1 and evaluates to a higher values for a larger number of training examples and a balanced number of positive and negative samples.

(9) $$\begin{array}{@{}rcl@{}} cellness(k) = (1-\nu) \cdot \sum \limits_{p\in \{1\ldots 5\}} \frac{1}{5} {s_{k}^{p}} + \nu \cdot {s_{k}^{6}} \end{array} $$

Figure 6c shows an example of the *cellness* confidence evaluation in a 3D volume of the ascidian *P. mammillata*. The colors in Fig. 6-c represent the degree of confidence (low to high: red-yellow-green). The red cell indicated by an arrow appears correctly segmented. However the reconstructed cross section in *x*−*z* reveals an error in the segmentation, as observed in Fig. 6d-e.

#### *CellECT* recommendations, segment propagation and cell analysis

In this section we discuss several features in *CellECT*, such as correction recommendations for the user, segmentation propagation to neighboring time points, and analysis tools for segmented volumes.

##### Recommendations

*CellECT* identifies problematic segments for the users to validate. Examples of such segments include spurious boundaries due to weak signal to noise ratio or dividing cells for which a nuclei detector may have discovered more than one nucleus. The mean intensity on the common boundary $\mathcal {B}_{\textit {kj}}$ of segment *k* and its neighbor *j* is given by $ \overline {\mathcal {B}_{\textit {kj}}}$. A merging score given by $\overline {\mathcal {B}_{\textit {kj}}} \cdot \frac {|\mathcal {B}_{\textit {kj}}|} { |\mathcal {B}_{k}|} $ is computed for every pair of neighboring segments. Pairs of segments are suggested to the user for merging or deletion in the increasing order of their scores.

##### Segment propagation

In order to facilitate high-throughput analysis *CellECT* allows users to propagate the segmentation results from one time point to the next. A simple approach is to transfer the background location and an interior point of each segment as seed points to the next (or previous) consecutive time point.

The segment inner point is given by the maximum of the distance transform (Eq. 3) from the segment boundary applied in the interior of the segment. Thus, the segment inner point is given by $\text {arg}\max \limits _{\mathbf {x} \in S_{k}} D_{\mathbf {x}}^{\mathcal {B}_{k}}$, where $\mathcal {S}_{k}$ denotes the interior of segment *k* and $\mathcal {B}_{k}$ denotes the border of segment *k*.

The new seeds will serve as input seeds to the new segmentation. In the event of errors in the resulting segmentation, the user can correct the segmentation by placing additional guidance seeds. Segmentations may be propagated using the interactive segmentation tool, or using the segmentation tool in non-interactive mode analysing a batch of time points together.

##### Cell analysis

*CellECT* includes an analysis tool, applicable to the multiple time-point segmentation results computed in the fashion described earlier. This tool can compute multiple local/global statistics as well as keep track of their changes over a subset of time points. Regions of interest may be selected for analysis by constraining position coordinates relative to the specimen.

Additionally, *CellECT* has a clustering module which implements K-means clustering algorithm. This module enables the user to cluster cells in a given volume based on similarity in a user-defined feature space. The user can select one or more features (Section “Segment features”) and specify the number of desired clusters for grouping cells. These parameters can be adapted to the data, and the resulting clusters can be visualized in the segmented volume. A number of group statistics (e.g. inter/intra-group variance, average cluster center distance etc.) are computed per volume over the time-series.

## Results and Discussion

Ascidians are used in the study of animal morphogenesis due to their small size, simple and compact embryo, and its similarity in early development to vertebrates. The Smith lab at UCSB uses microscopy volumes of ascidians for quantitative analysis in morphogenesis research [19, 20].

Two 3D+t datasets of the ascidian *P. mammalitta* are analyzed using *CellECT*. The first dataset, *Ascidian-18*, is a confocal microscopy time series which consists of 18 time points (26 slices per volume), from stage 15 to stage 21 [21] with membrane and nuclei channels. This dataset starts out with approximately 300 cells which develop into 500 cells. The second dataset, *Ascidian-192*, is a SPIM time series which consists of 192 time points (197 slices per volume), from stage 6 to stage 19, also with membrane and nuclei channels. This dataset starts out with 32 cells which develop into almost 1000 cells.

Additionally, a third case study from a different application is considered: The leaf and cotyledon epidermal cells of dicot plants are highly interdigitated with a jigsaw-puzzle piece shape. Using *Arabisopsis* as a model, it has been shown that the growth properties of the epidermis influence the size and shape of the organ [22]. Therefore, understanding how the growth properties of the cell relate to organ form is an important biological question. Historically, measurements of these cells have been done by manually segmenting each cell [23–26], a highly time-consuming procedure, but recently there has been a push for a more automated approach [27]. The *A. thaliana* dataset consists of 112 individual cells from 2D confocal microscopy slices which were segmented using *CellECT*.

### Ascidian *P. mammalitta* - 18

**Fig. 7:**
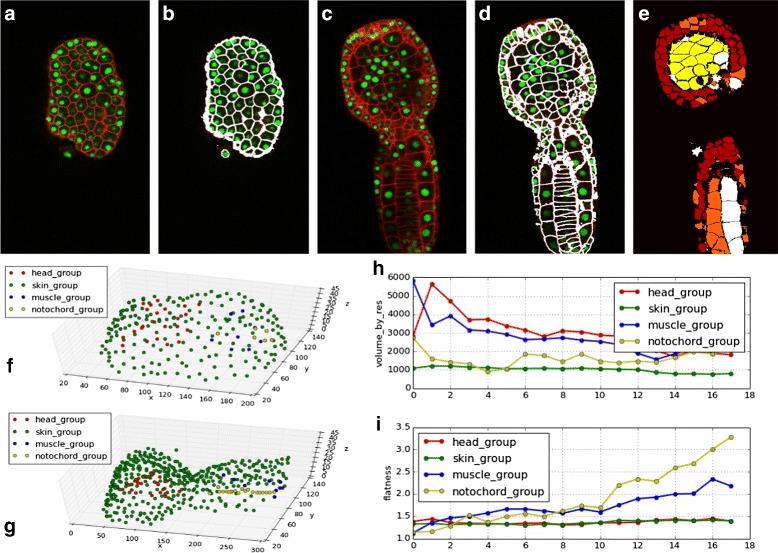
*Ascidian-18* dataset: **a**–**d** original slice and respective segmentation for *t*=0 (stage 15) and *t*=17 (stage 21) (E) Clustering of cells with similar properties identifies tissues **f – g** Nuclei at *t*=0 and *t*=17 in each of the four regions of interest: notochord (*yellow*), muscle (*blue*), endoderm and neural tube (*red*), epidermis (*green*). **h**–**i** Average cell measurments over time per region of interest: **h** volume **i** flatness

The method in [10] is used to detect nuclei. Segmentation results corresponding to the first and last time points are presented in Fig. 7a-d. *CellECT*’s analysis module is used to isolate several regions of interest and observe changes over the development of the embryo. This datasets captures the development of the embryo up to tailbud stage 21 [21]. The following regions of interest are relevant at the tailbud stage: the notochord tissue forms in the posterior half of the embryo along the AP axis; muscle cells develop surrounding the notochord tissue; the epidermis develops at the surface of the embryo; finally, the cells in the dorsal half of the embryo, below the epidermis, represent the neural tube. These four regions of interest are marked by restricting the spatial properties computed in Section “Segment features”. Figure 7f-g shows the spatial arrangement of the cell nuclei color coded by their membership to one of these regions of interest.

Statistics over various cell measurements in each of these regions of interest are computed. For example, Fig. 7h-i shows the average volume and cell flatness over the 18 time points in each region. As expected from the known development of the ascidian tailbud, notochord cells become mostly flat, followed by muscle cells. Also, as a result of cell divisions the average volume over time decreases, while muscle cells and endodermal cells maintain the highest volume. These measurements confirm the expected developmental behavior, suggesting that the segmentation label maps resulting from *CellECT* are accurate.

Additionally, segments are clustered in feature space in order to group similar cells using K-means clustering algorithm [28]. The inter-cluster distance was computed for every time point, and an increasing trend was observed (from 2.6 units in the normalized feature space to 3.6 units). This is due to the fact that cells specialize as they form tissues. These measurements meet the expected behavior, suggesting that the segmentation label maps resulting from *CellECT* are reliable. An example of such clustering is shown in Fig. 7e, where the color coding marks each of the four clusters.

### Ascidian *P. mammalitta* - 192

**Fig. 8:**
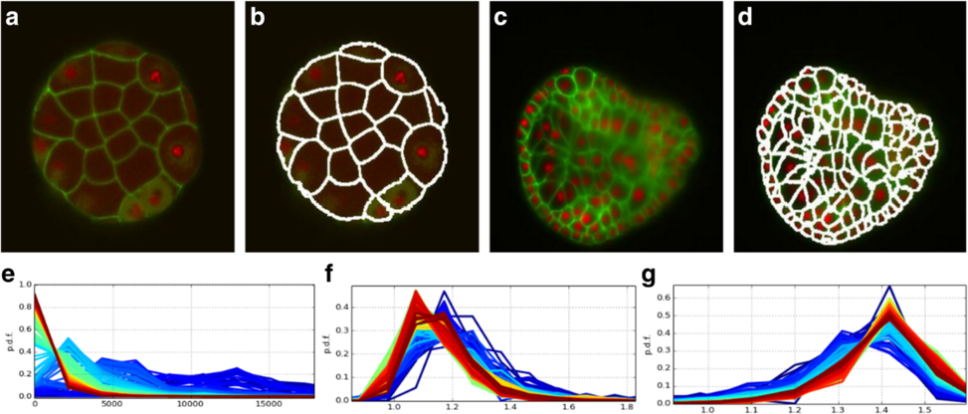
*Ascidian-192* dataset: **a**–**d** original slice and respective segmentation for *t*=0 (stage 6) and *t*=192 (stage 19) **e**–**g**: Superimposed histograms of segment measurments, color coded by time point (from *blue* to *red*): **e** volume, **b** sphericity, **c** entropy

The nuclei detector of [10] did not perform well on this dataset due to artifacts such as those in Fig. 1a, f. Only about 500 out of the approximately 900 cells in the last time point were detected. *CellECT*’s interactive segmentation tool is used to correct and propagate the segmentation results. The interactive recommendation feature in the segmentation module was used to quickly identify the dividing cells. Figure 8a-d shows a slice through the first and the last time points of the dataset and their respective segmentation.

This dataset starts out with 32 cells in the first time point and ends with approximately 900 cells in the last time point. Using *CellECT* analysis module various measurements are computed over the time sequence. Figure 8e-g shows three of these measurments over time: the histogram of volume of cells at each time point, the histogram of sphericity values over time and the histogram of entropy values over time. Each histogram is color coded by the time point it represents. As expected, cells exhibit lower surface area in the later time points. This is a result of cell division resulting in many more cells occupying approximately the same total volume as the early time points. Similarly, cells in later time points exhibit lower entropy, suggesting that cell shape become more compact over time.

### Arabidopsis pavement cells

Images were collected using a scanning confocal microscope with a 40X-oil immersion objective [29]. This dataset lacks a nuclear marker to automatically identify each cell. Cell identification is assigned manually and segmentation is based on fluorescent signal of the lipophilic dye FM4-64 which labels cell periphery. Cell segmentation is restricted to pavement cells that are completely contained within the image field. The same cells are also segmented manually and morphometric measures (area, perimeter and circularity) are obtained on both sets. These measures are compared against each other to determine the quality of the segmentation in Section “Quantitative evaluation of segmentation quality using *A. thaliana* slices”. Small symmetrical cells associated with the stomatal lineage are also accurately segmented using CellECT, but are not included in the morphometric analyses.

**Fig. 9:**
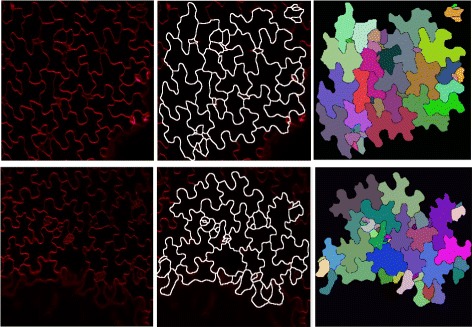
*A. thaliana* pavement cells: *Left column*: Original slices, *Middle*: Segmentations overlaid on original slices, *Right*: Segmentation label maps

*CellECT* reduced the necessary time for cell segmentation by a factor of ten when compared to manual methods. Two examples of confocal slices and the respective segmentations are shown in Fig. 9.

### Analysis

Next, we evaluate the quality of the segmentations obtained using *CellECT* and the efficiency in using the *cellness* metric.

#### Quantitative evaluation of segmentation quality using *A. thaliana* slices

The segmentations obtained in Section “Arabidopsis pavement cells” are compared against manually obtained segmentations in order to evaluate the segmentation quality. XY-coordinates were extracted from *CellECT*’s results and imported as a ROI into the scientific image viewing software, ImageJ [30]. Manual segmentation was conducted on the same cells segmented by *CellECT* using the polygon selection tool in ImageJ with the spline function active.

A total of 112 pavement cells were segmented from three different plants all taken at the same developmental stage. The majority of the cells in the field were accurately segmented, and an average of 14 pavement cells from 5 day after germination (DAG) cotyledons were obtained from an image field of 308 × 308 *μ*m. Measurements of cell area, perimeter, and circularity (unitless shape descriptor), which are commonly used for pavement cell analyses, were taken from cell outlines that were manually segmented and those obtained using *CellECT*. Cell population from each image field were tested for normal distribution and analyzed using either Student’s t-test for populations with normal distribution or Mann-Whitney test for populations with non-normal distributions. A *p*-value less than 0.05 indicates a statistical significant difference between parameter outputs from *CellECT* and manual segmentation Table 1.

**Table 1:** *A. thaliana* pavement cells analysis: *p*-value for Student’s t-test analysis between *CellECT* and manual segmentation

Dataset	n	Area	Perimeter	Circularity
8452C2F2	9	0.758	0.846	0.922
8453C1F1	12	0.672	0.783	0.969
8453C2F1	7	0.764	0.901	0.955
8453C2F2	24	0.533*	0.493*	0.901
8453C2F3	19	0.704	0.822	0.986
8454C2F2	11	0.585	0.714	0.828
8455C2F2	6	0.775	0.851	0.870*
8456C1F1	24	0.403	0.522	0.628*

^*^Mann-Whitney test performed

#### Quantitative evaluation of segmentation quality and *CellECT*’s segmentation propagation feature using the *Ascidian-192* dataset

In this section we compare the segmentations obtained using *CellECT*’s workflow against manually generated 3D ground truth for a subset of cells. We compare different approaches in order to determine the benefits of the interactive segmentation feature.

To demonstrate the utility of propagation over consecutive time points, a set of fifty cells spanning over five consecutive time points was manually traced in 3D. Ten cells per time point were selected for the last five time points of the *Ascidian-192* dataset and ground truth segmentation was generated. This set contains cells from different tissues, as well as special cases such as dividing cells. The metric used for comparison is the *F*-measure which is a volume based error metric defined as

(10) $$ F = \frac{2 \cdot P \cdot R} {P + R}  $$

where *P* and *R* are the precision and recall of the corresponding ground truth volume.

In order to evaluate the utility of the segmentation tool, four different segmentation approaches were compared, and the *F*-measure was evaluated for every cell for which ground truth is available. The *F*-measure corresponding to the fifty segmented cells is shown in Fig. 10a, where the *x*-axis corresponds to the index of the fifty cells. Cells 1–10, 11–20, 21–30, 31–40 and 41–50 correspond to time points 188, 189, 190, 191 and 192, respectively. Four different approaches to the segmentation of these five consecutive volumes were explored:

1. First, the volumes were segmented by initializing the algorithm with the output of the nuclei detector of [10]. This dataset is particularly challenging for a nuclei detection algorithm as shown in Fig. 1a and Fig. 1f. As seen in Fig. 10a this initialization results in the incorrect segmentation of several cells. Using the interactive segmentation tool, the initialization errors can be corrected and subsequently propagated to neighboring time points, which in turn may be corrected in the event of any additional mistakes. These workflows are explored next.

**Fig. 10:**
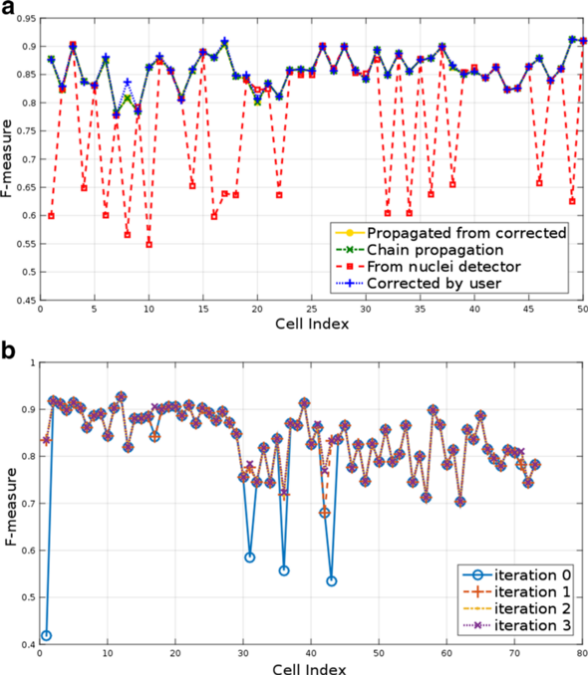
Quantitative evaluation of the segmentation: **a** *F*-measure for the segmentation of fifty randomly selected cells from the last five time points of the *Ascidian-192* dataset. Four segmentation results are compared: (1) nuclei detector initialization, (2) propagation of corrected volumes, (3) chain propagation without corrections, (4) propagated and corrected volumes. **b** *F*-measure for four approaches to the segmentation of time points 188–192 from the *Ascidian-192* dataset
2. The second approach consists of correcting the last time point (cells with index 41–50) and propagating this corrected segmentation to the previous four time points (cells 1–40). Thus a chain of propagated segmentations is obtained without any user intervention, except for the last time point.
3. The third approach measures the quality in segmentation when every propagated volume is corrected for potential errors. This approach evaluated the quality of a segmentation propagated from a corrected result and before any additional human interaction.
4. Finally, the fourth approach measures the quality of segmentation when propagating from a corrected volume and after correcting any resulting errors.

As seen in Fig. 10a, there are little differences in the scores of the three propagation approaches and they compare favorably to the results without any human intervention. Therefore, propagating a corrected volume reduces the need for human intervention. The average *F*-measure for each of the approaches is listed in Table 2. In conclusion, the interactive segmentation tool coupled with the propagation feature enables segmentation with reduced human interaction of large time sequences for which automated methods often have difficulty. For example, in Fig. 10a, several cells obtained scores as low as 55 % with automated nuclei detection, but instead, using *CellECT*’s features, a correct segmentation can be propagated to subsequent time points. As a result, *F*-scores above 80 % can be obtained without any additional user interaction.

**Table 2:** Average *F*-measure for four approaches to the segmentation of time points 188–192 from the *Ascidian-192* dataset

Approach	Avg *F*-measure
(1) Nuclei detector initialization	0.7789
(2) Chain propagation, no corrections	0.8570
(3) Propagation from corrected, no corrections	0.8570
(4) Propagation from corrected, with corrections	0.8582

Next, we use 73 cells from the first time point of the *Ascidian-18* dataset for which manually traced 3D ground truth is provided. We observe improvements in segmentation over multiple user feedback iterations using the *F*-measure for each cell. As seen in Fig. 10b the problematic cells are corrected and a satisfactory segmentation is obtained in four iterations. The average *F*-measure at each iteration is listed in Table 3, where the first iteration starts out with the output from the nuclei detection algorithm. The most significant corrections are made in the early iterations.

**Table 3:** Average *F*-measure over four iterations for the segmentation of the first time point of the *Ascidian-18* dataset

Iteration	Avg *F*-measure
0	0.8223
1	0.8370
2	0.8395
3	0.8398

#### Quantitative evaluation of cellness metric performance using *Ascidian-18* dataset

In this section we investigate if the cellness metric can effectively identify incorrect segments. The following experiments are performed using segmentations from the *Ascidian-18* dataset. In the first experiment we compare the *cellness* metric score for two sets of cells which are manually annotated into one of two classes: “Correct” and “Incorrect”. An effective *cellness* metric is expected to show a distinct separation between the two classes. This experiment is performed on five time points of the dataset, where approximately 15–20 cells of each class are selected at each time point. In a second experiment, ten “Correct” and ten “Incorrect” cells are selected from the final time point, for which each component of the *cellness* score is observed.

**Fig. 11:**
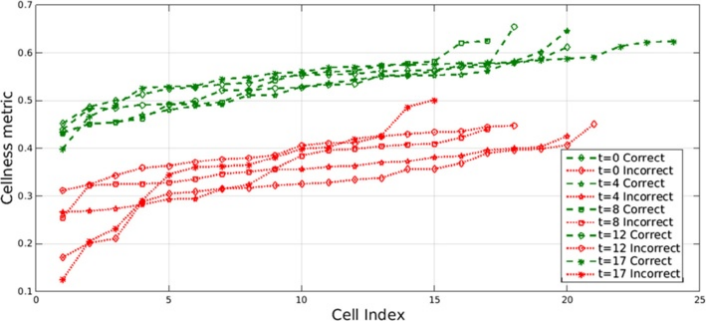
Cellness metric for correct and incorrect cells. Cellness metric in sorted order for hand picked cells in two categories, “Correct” and “Incorrect”, over five time points (*t*=0,4,8,12,17) of the *Ascidian-18* dataset. The two classes of cells separate well for the different time points

In the first experiment, we manually identify 15–20 correctly segmented cells from each of the time points *t*=0,4,8,12,17. Similarly 15–20 incorrectly segmented cells are manually selected from each of these five time points. The *cellness* metric for the cells in these two categories is plotted in sorted order in Fig. 11. The two categories of cells separate well for every time point. The average *cellness* metric for the “Correct” and “Incorrect” groups for these time points is listed in Table 4. As expected, “Correct” cells consistently obtain a higher *cellness* metric than the set of “Incorrect” cells in each time point.

**Table 4:** Average *cellness* metric for 15–20 cells from each of the time points *t*=0,4,8,12,17 from two hand picked categories: “Correct” and “Incorrect” cells

Time point	*Cellness* “Correct” cells	*Cellness* “Incorrect” Cells
0	0.5468	0.3281
4	0.5253	0.3443
8	0.5298	0.3653
12	0.5289	0.3924
17	0.5576	0.3539

**Fig. 12:**
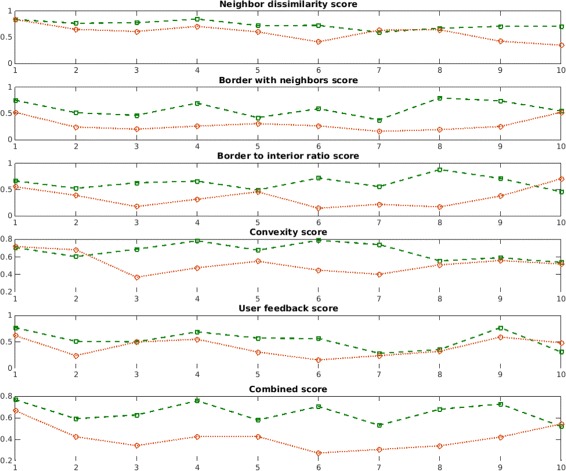
Cellness metric components. Score of each *cellness* metric component over ten “Correct” and ten “Incorrect” cells from *t*=17 of the *Ascidian*-18 dataset, and the combined *cellness* score

**Fig. 13:**
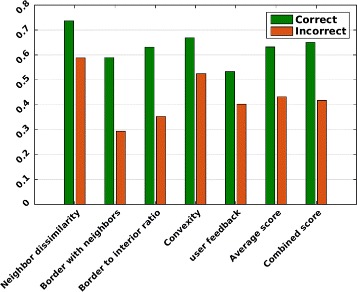
Average cellness metric components. Average scores over the ten cells in each class for each *cellness* metric component, the average of all components, and the combined score (*cellness*). “Correct” cells obtain a higher *cellness* score than “Incorrect” cells

Next, in the second experiment, we examine the contribution of each component of the *cellness* metric. Ten “Correct” cells and ten “Incorrect” cells are selected from the last time point of the dataset. Fig. 12 shows the contribution of each *cellness* score for every cell of the “Correct” and “Incorrect” categories: ${s_{k}^{1}}$, ${s_{k}^{2}}$, ${s_{k}^{3}}$, ${s_{k}^{4}}$ and ${s_{k}^{5}}$ and *cellness*(*k*) described in Section “Learning a cellness metric”. In addition, Fig. 13 and Table 5 show the average score for each *cellness* component over the ten cells of each category. This experiment demonstrates that the *cellness* metric effectively captures cues which indicate the quality of the segmentation.

**Table 5:** Average score for every *cellness* component of ten “Correct” cells and ten “Incorrect” cells

Score component	“Correct” cells	“Incorrect” Cells
Neighbor similarity	0.7365	0.5878
Border with neighbors	0.5890	0.2933
Border to interior ratio	0.6307	0.3526
Convexity	0.6684	0.5246
User feedback	0.5330	0.4018
Average of score components	0.6315	0.4320
Combined score (*cellness*)	0.6498	0.4173

## Conclusion

We introduced a software for the interactive segmentation and analysis of 3D+t membrane or cell wall image datasets. *CellECT* enables users to create and interact with the segmentation of images containing cell boundary information by adding, deleting, or modifying segments. An adaptive confidence metric (*cellness* metric) helps identify areas of uncertain segmentation. The algorithm is able to identify spurious boundaries and suggest corrections. Segmentation results can be propagated to neighboring time points. Once segmentation is obtained for multiple consecutive time points the analysis tool displays statistics over time and allows the user to focus on regions of interest.

We demonstrated to utility of this framework by quantitatively evaluating the quality of segmentations and the efficiency of the *cellness* metric. Case study analysis was performed on three datasets: a time series of 18 volumes of the of the Ascidian *P. mammillata*, a time series of 192 volumes of the same species, and a set of 112 cells from 8 confocal slices of *A. thaliana* pavement cells. Cells from different time points of the two *P. mammillata* datasets were compared against manually segmented cells. Additionally, the efficiency of *CellECT*’s segmentation propagation feature and the utility of the *cellness* metric were demonstrated in quantitative analysis. In the case of the *A. thaliana*, *CellECT* reduced the segmentation time by a factor of 10 when compared to manual methods without reducing the quality of segmentations. No statistical significant differences were found between *CellECT* coordinates and manually extracted cells in the parameters of area, perimeter, and circularity.

Future work aims at developing a cell lineage reconstruction module. An integrated framework which jointly detects nuclei, computes cell segmentation and reconstructs lineage over the time series in a continuously adaptive feedback loop is desired. Additional future plans include the integration with *BISQUE*, introduced in [31], an online web-based bio-image analysis system which facilitates collaboration among biologists.

## Availability of supporting data

*CellECT* is an open source project available at http://bioimage.ucsb.edu/. Supplementary material is available such as demo video (Additional file 1), detailed results (Additional file 2) and animations (Additional files 3-9).

## Additional files

Additional file 1
***CellECT*** **Demo.** Video demonstrating the utility of *CellECT*. (MP4 53900 kb)

Additional file 2
**Additional analysis.** Additional plots and measurements obtained on *Ascidian-18* and *Ascidian-192* datasets. (PDF 4850 kb)

Additional file 3
**Ascidian-18 middle slice first to last time point.** Animation showing the middle slice of the *Ascidian-18* dataset from the first to the last time point. (GIF 4640 kb)

Additional file 4
**Ascidian-18 middle slice first to last time point.** Animation showing the middle slice of the segmentation of the *Ascidian-18* dataset from the first to the last time point. (MP4 1790 kb)

Additional file 5
**Ascidian-18 first time point.** Animation showing the first time point of the *Ascidian-18* dataset. (MP4 1270 kb)

Additional file 6
**Ascidian-18 first time point segmented.** Animation showing the segmentation of the first time point of the *Ascidian-18* dataset. (MP4 1290 kb)

Additional file 7
**Ascidian-18 last time point.** Animation showing the last time point of the *Ascidian-18* dataset. (MP4 986 kb)

Additional file 8
**Ascidian-18 last time point segmented.** Animation showing the segmentation of the last time point of the *Ascidian-18* dataset. (MP4 970 kb)

Additional file 9
**Cells clustered by features.** Animation showing cells clustered by features in the last time point of the *Ascidian-18* dataset. (GIF 400 kb)
