# Supplementary material for: CellECT: cell evolution capturing tool
Source: BMC Bioinformatics. 2016 Feb 17;17:88. doi: 10.1186/s12859-016-0927-7 (PMC4756481; doi:10.1186/s12859-016-0927-7)

This document contains supplementary material for the manuscript submission titled "CellECT: Cell Evolution Capturing Tool" by Delibaltov et al. to BMC Bioinformatics.

1. **Screenshots of CellECT software**
2. **Supplementary analysis for "Ascidian-18" dataset**
3. **Supplementary analysis for "Ascidian-192" dataset**

1. Screen Shots

Main window, after having loaded a dataset and before segmentation was computed. User can slide through time points (list on the left), can append additional time points, or start interactive (or automated) segmentation.

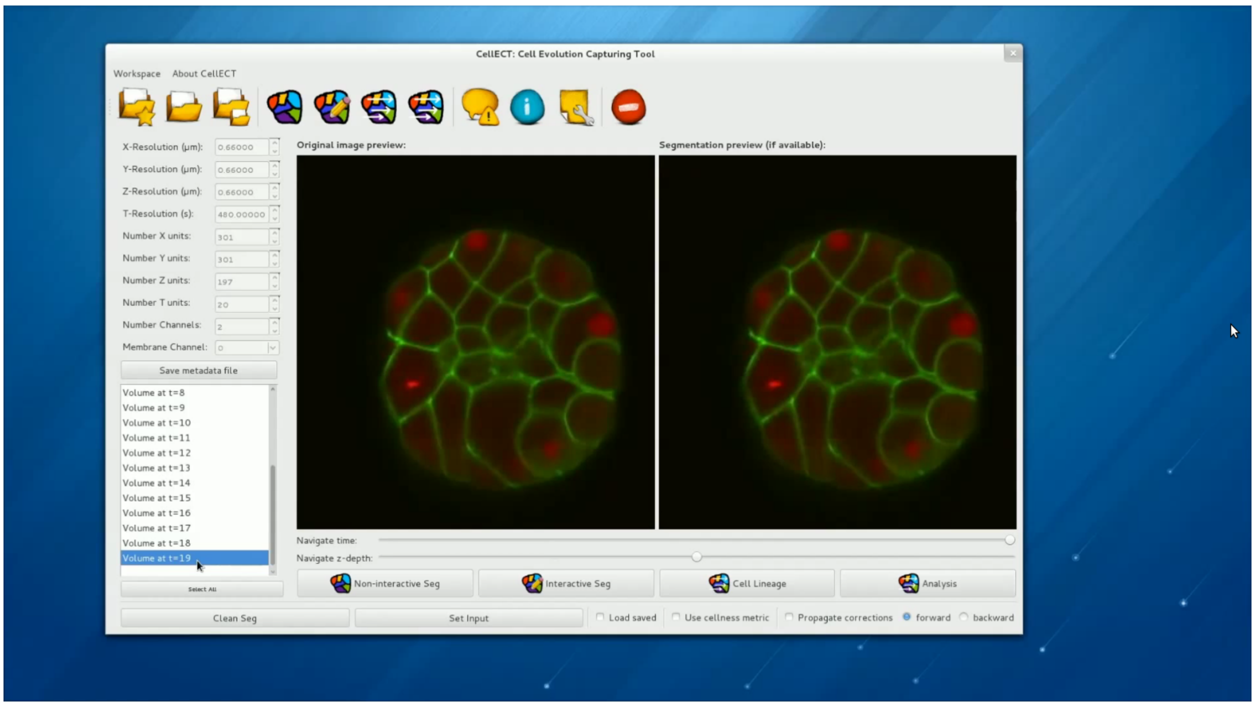

Example workspace which contains segmentation. User may modify the available segmentation or analyze results.

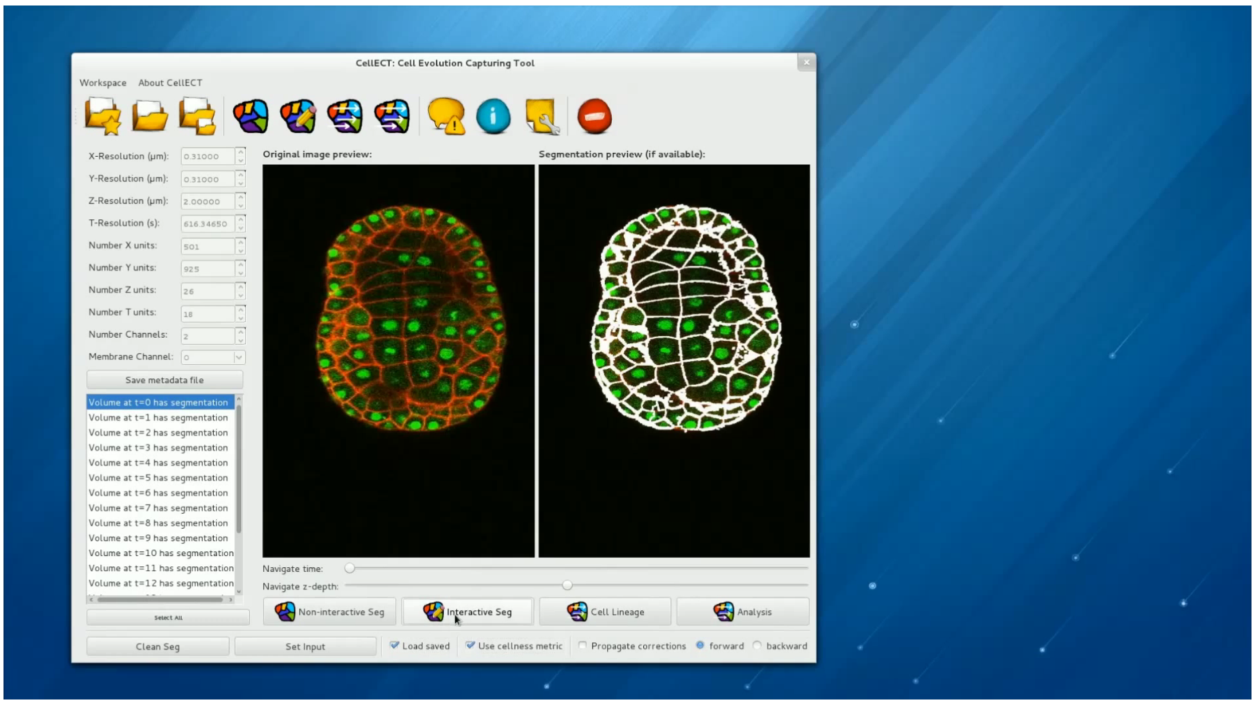

Example interface for interactive tool. Four panels from left to right: slice through microscopy image, segments color coded by cellness metric, segments randomly color coded, difference map which shows which segments were modified from the previous iteration. The user may select a segment based on the cellness metric and correct it with guidance seeds.

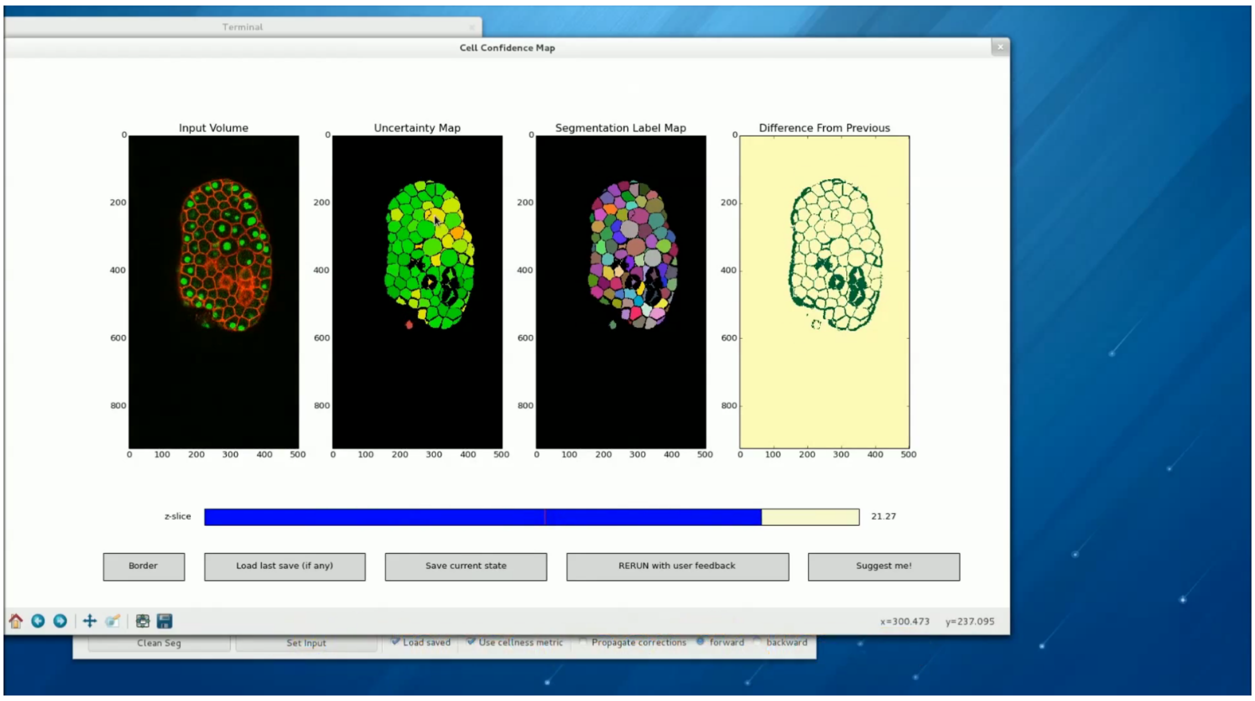

Example interface for correcting a segment, where the user may place guidance seeds to modify, add, merge or remove segments.

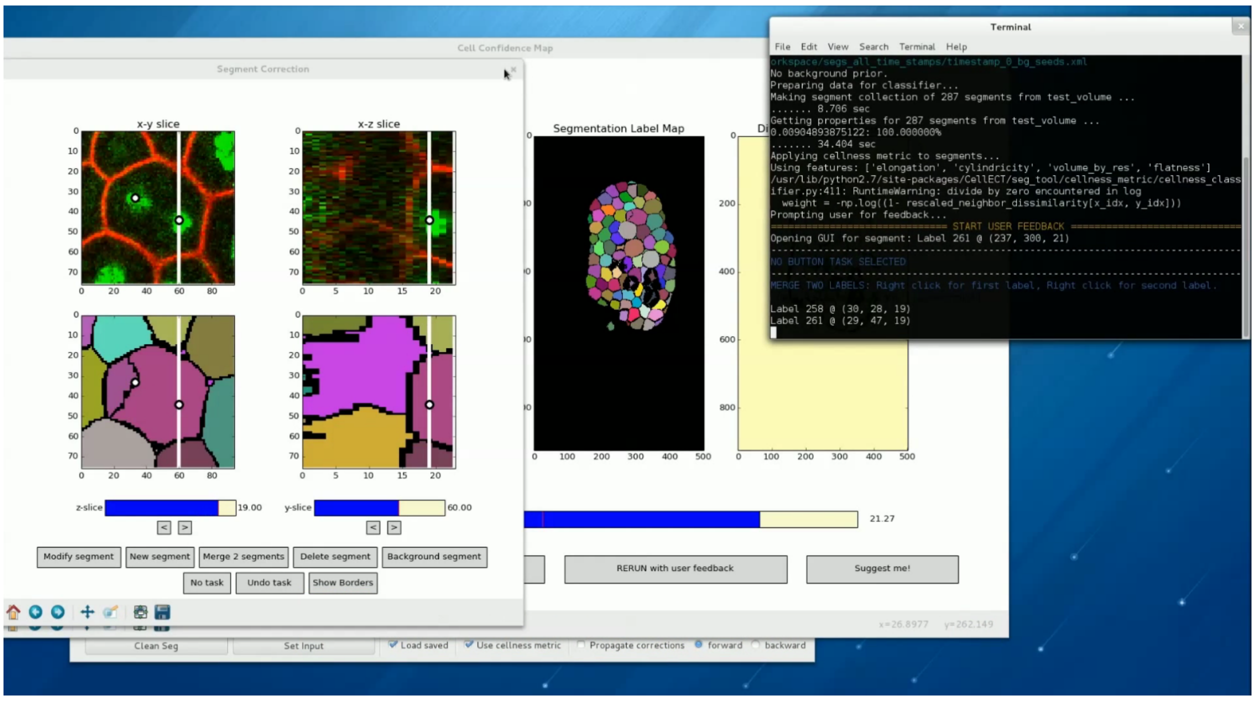

Example interface for “merge segments” suggestions. The user may ask for suggestions from the algorithm. The two cells colored in yellow and red are suggested for merging.

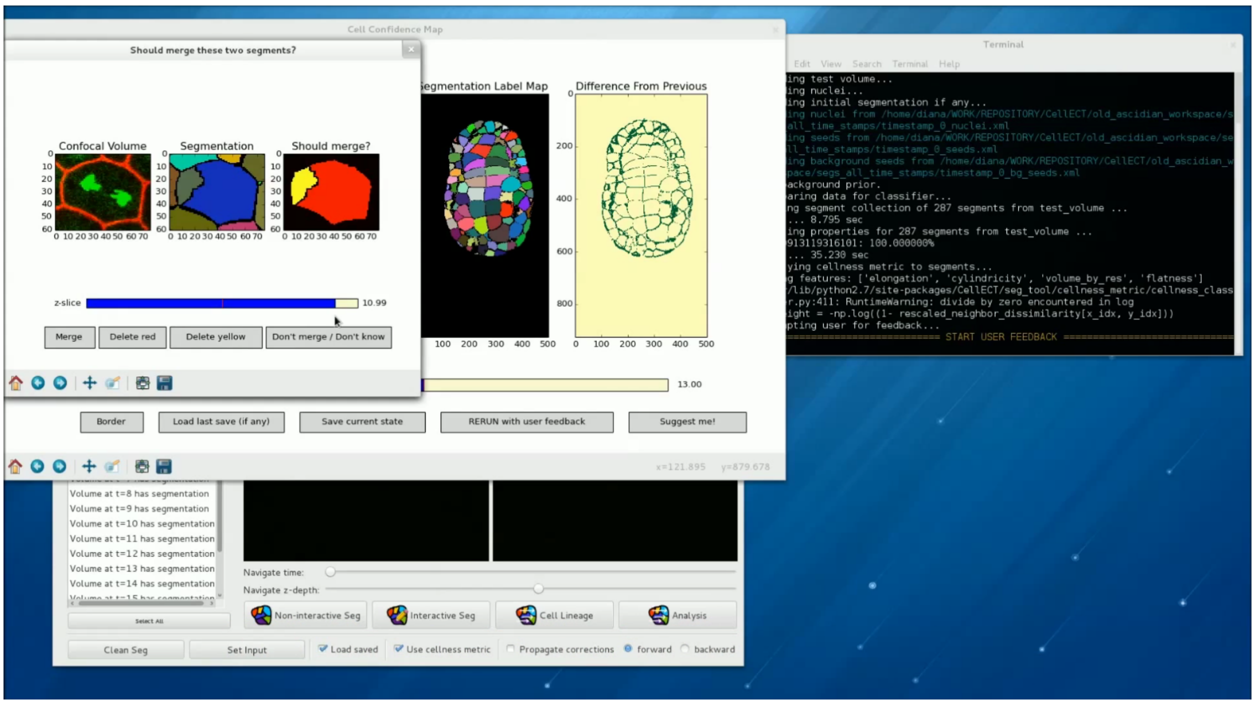

Once segmentation on multiple time points is available the trends in segment measurements over time may be computed.

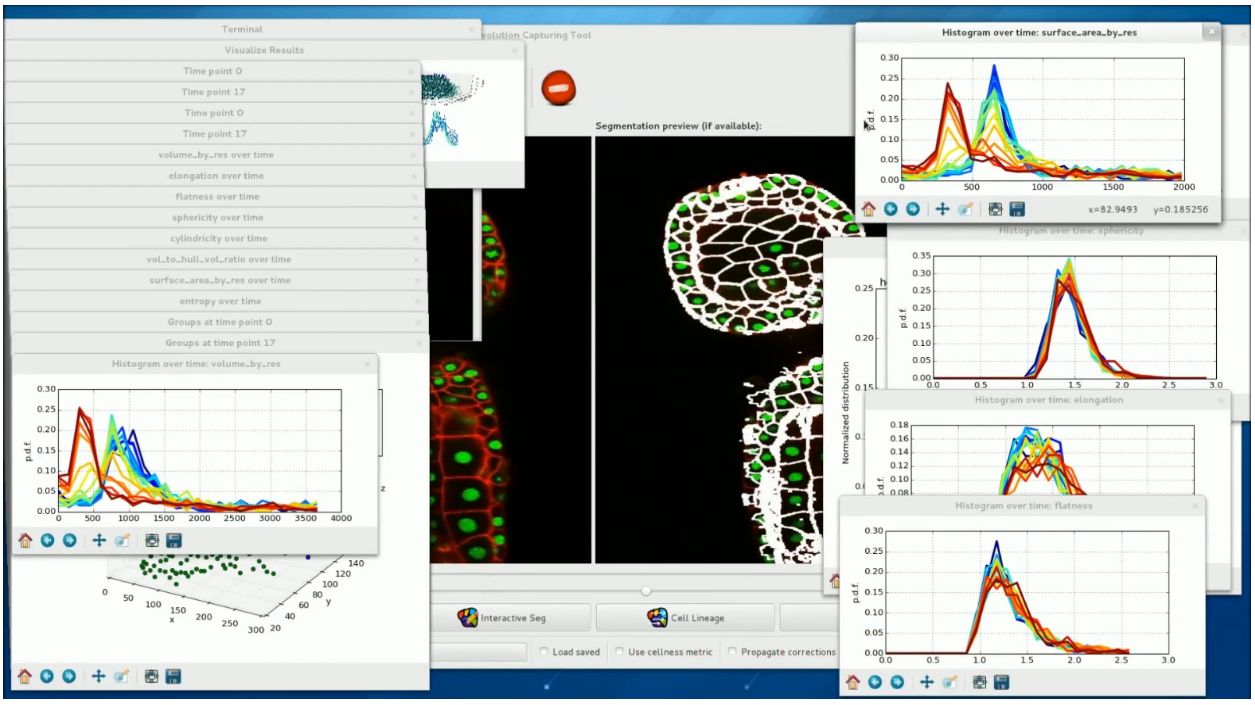

2. Ascidian-18 Analysis

Segment shape measurements over time in Head, Skin, Muscle, Notochord regions of interest.

Flatness Vs Volume at the first time point.

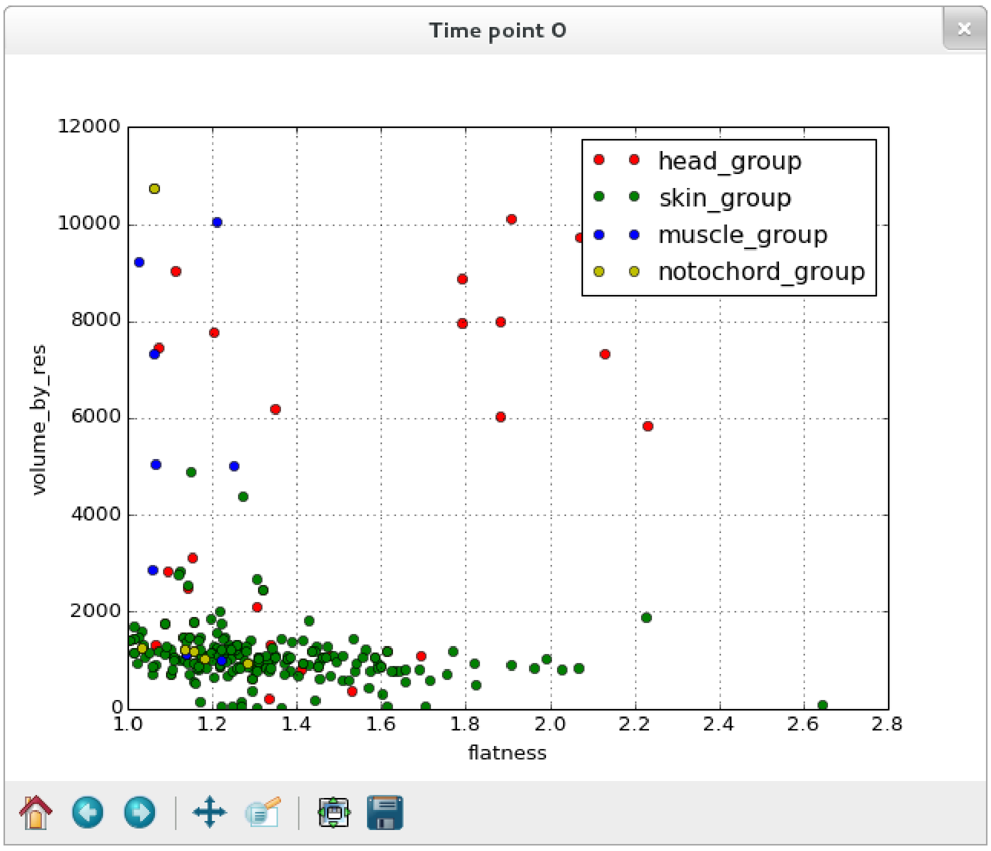

Flatness Vs Volume at the last time point.

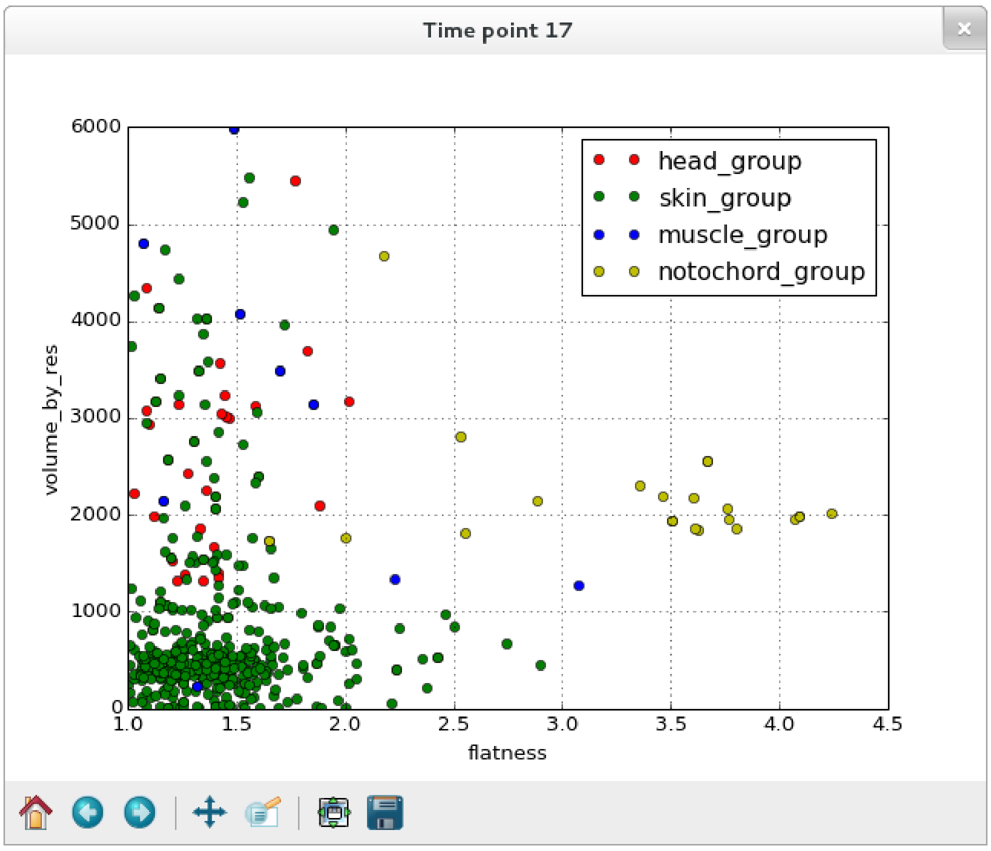

Elongation Vs Volume at the first time point.

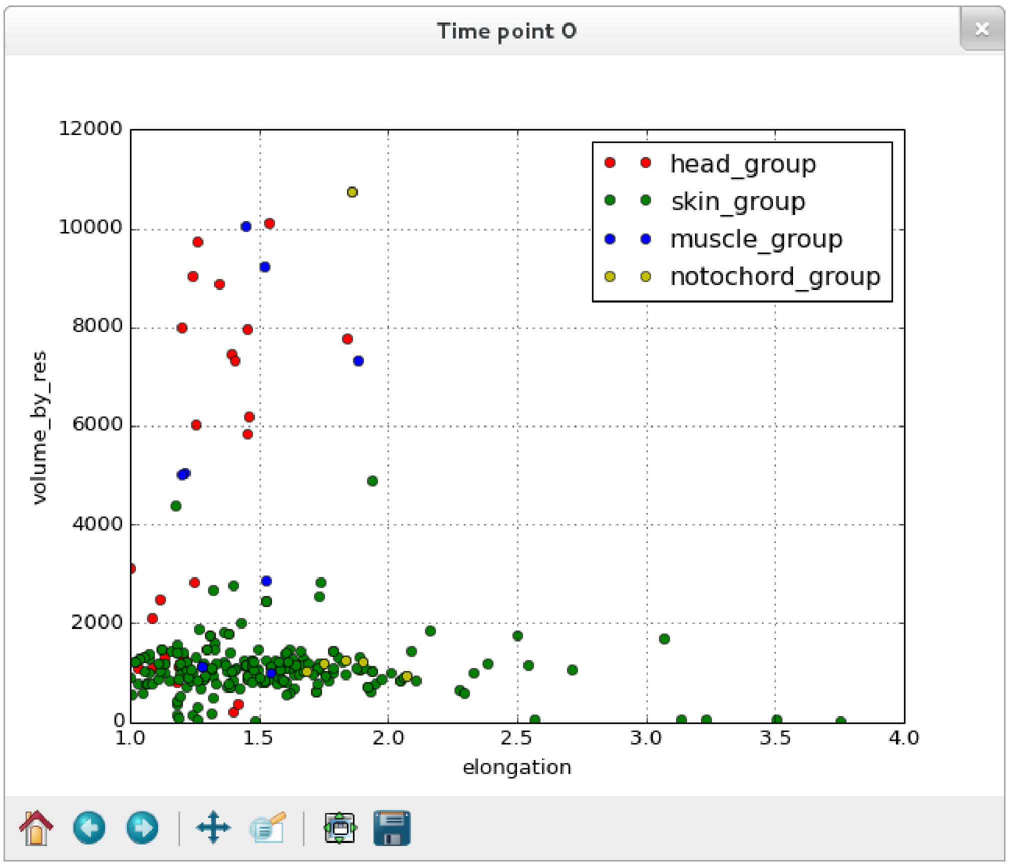

Elongation Vs Volume at the first time point.

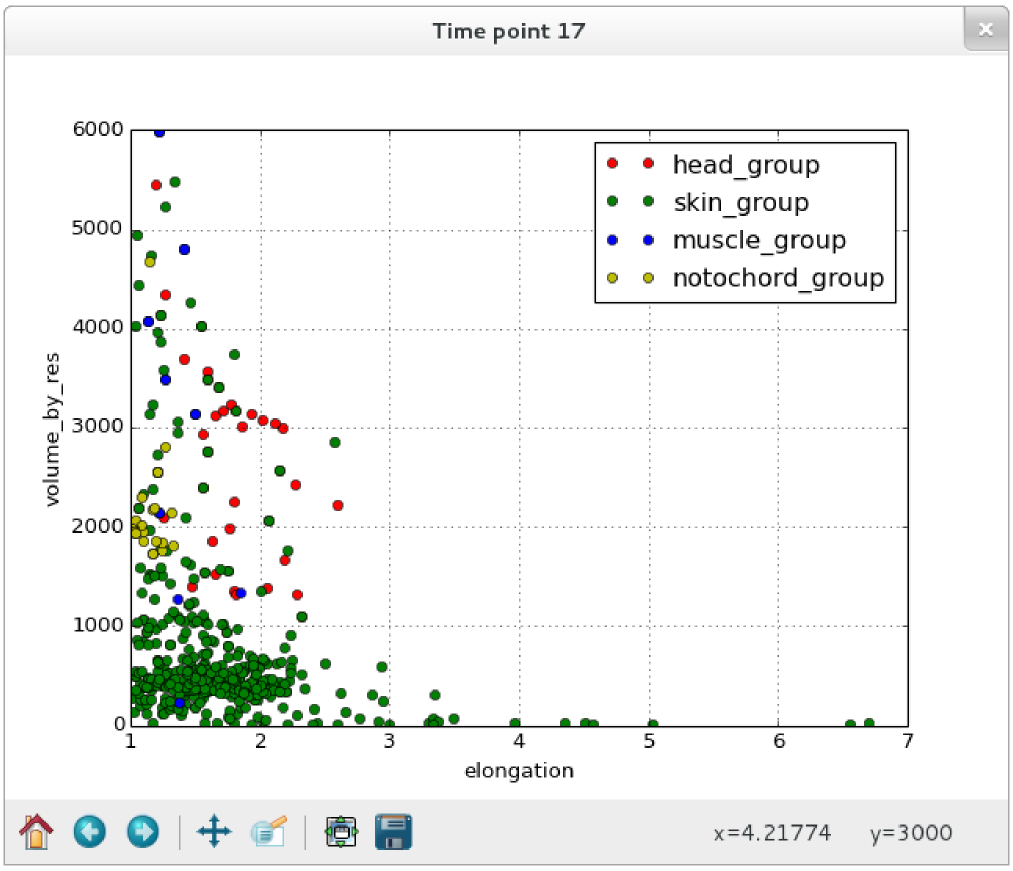

Sphericity over time in each region of interest. Low values indicate more spherical.

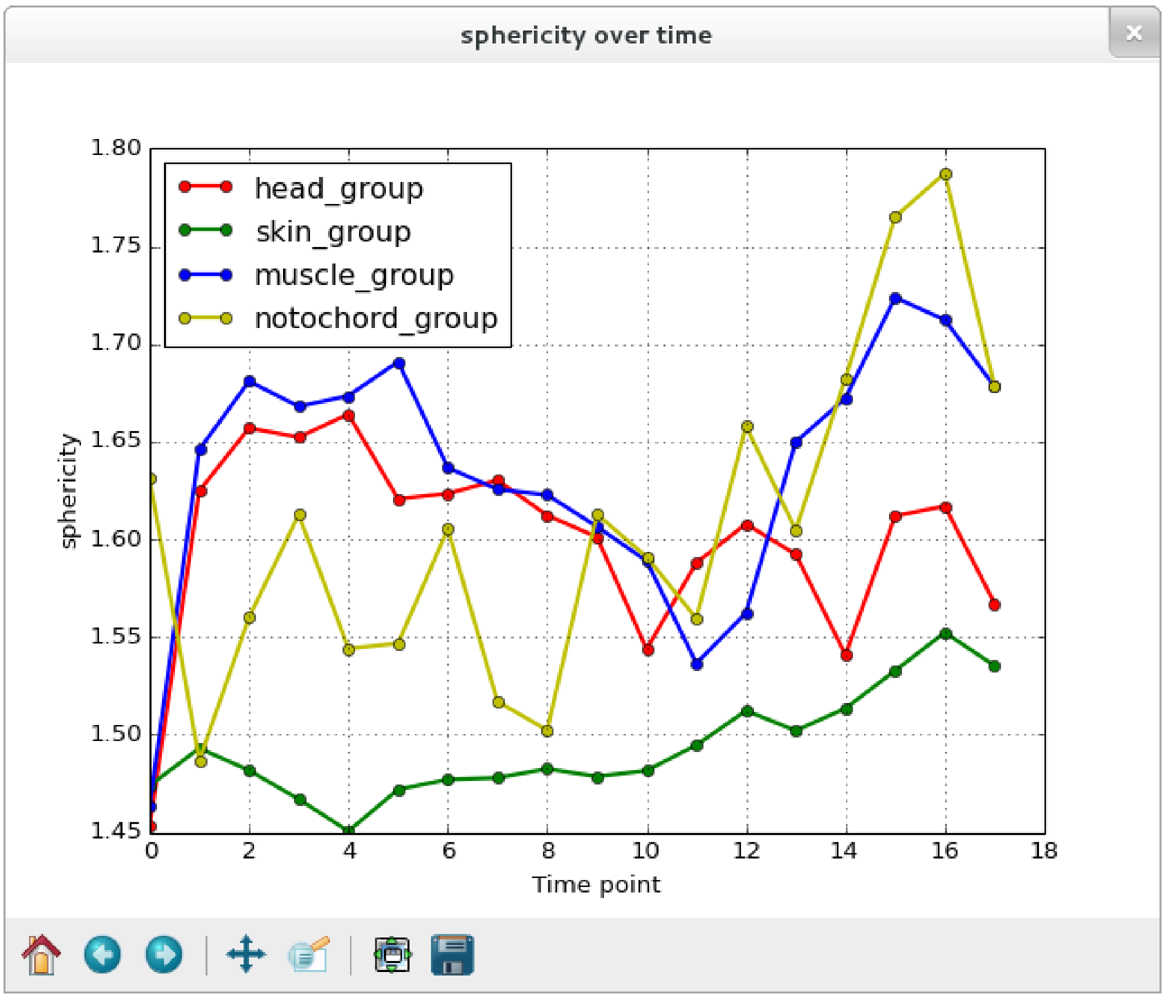

Volume over time in each region of interest.

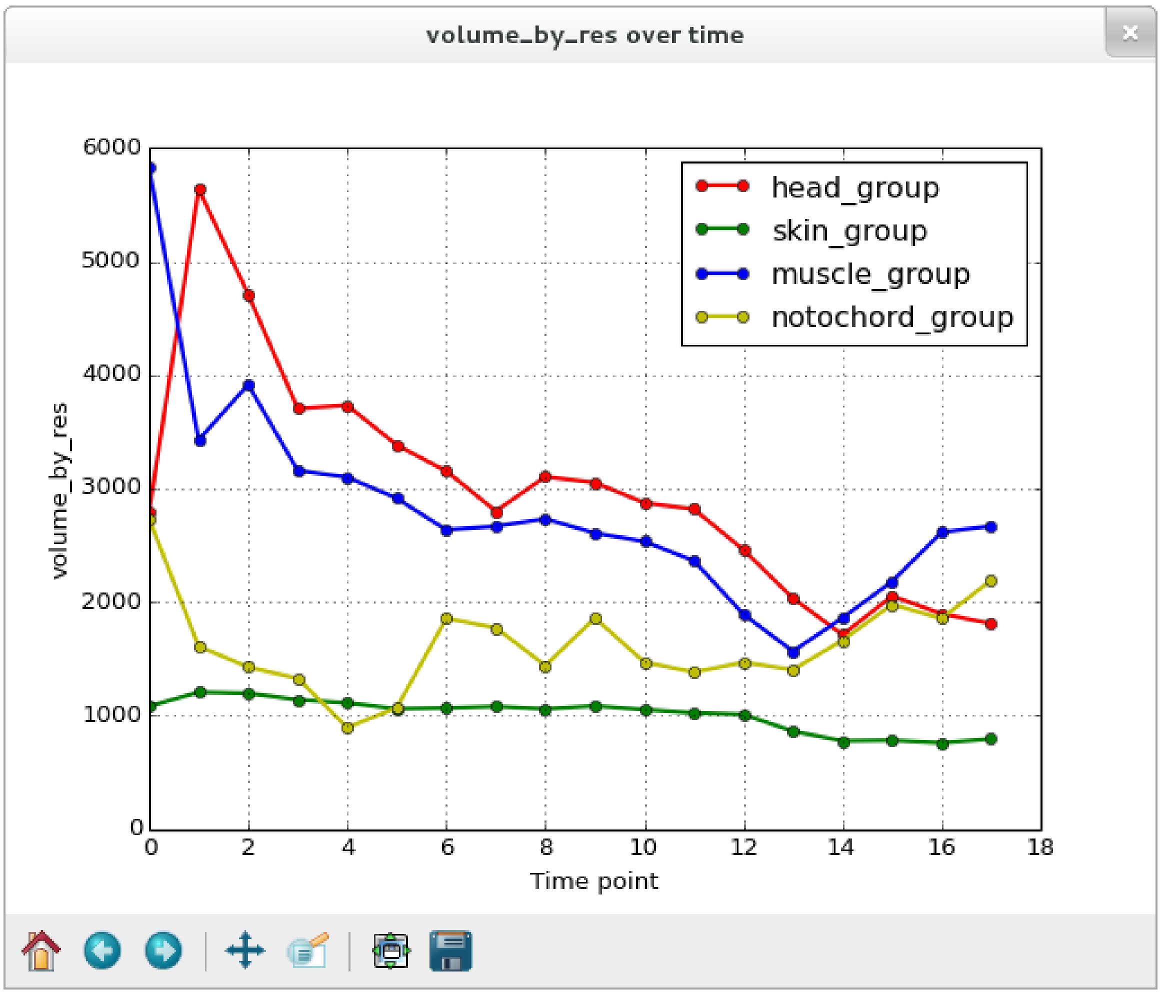

Elongation over time in each region of interest. High values indicate more elongated cells.

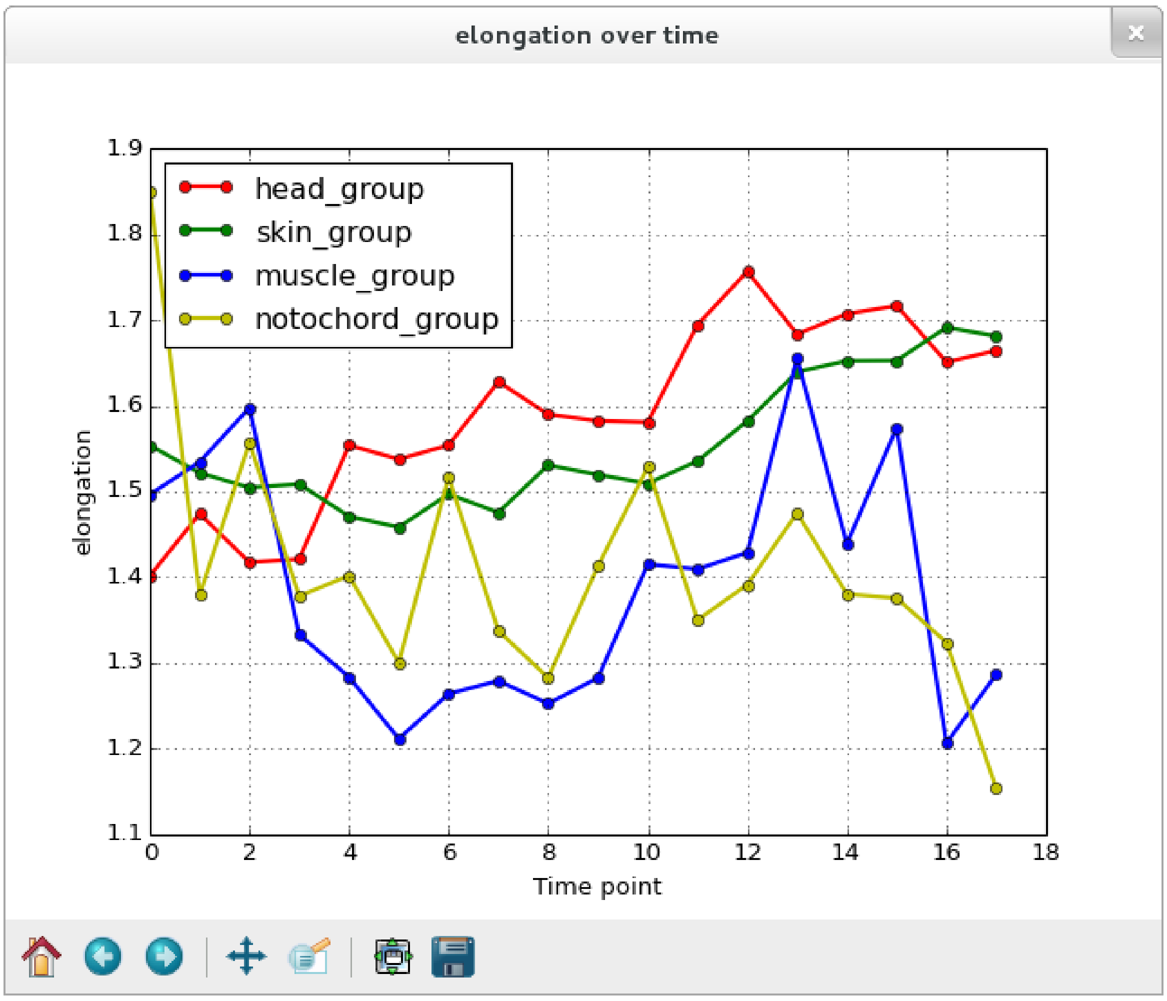

Flatness over time in each region of interest. High values indicate flatter cells.

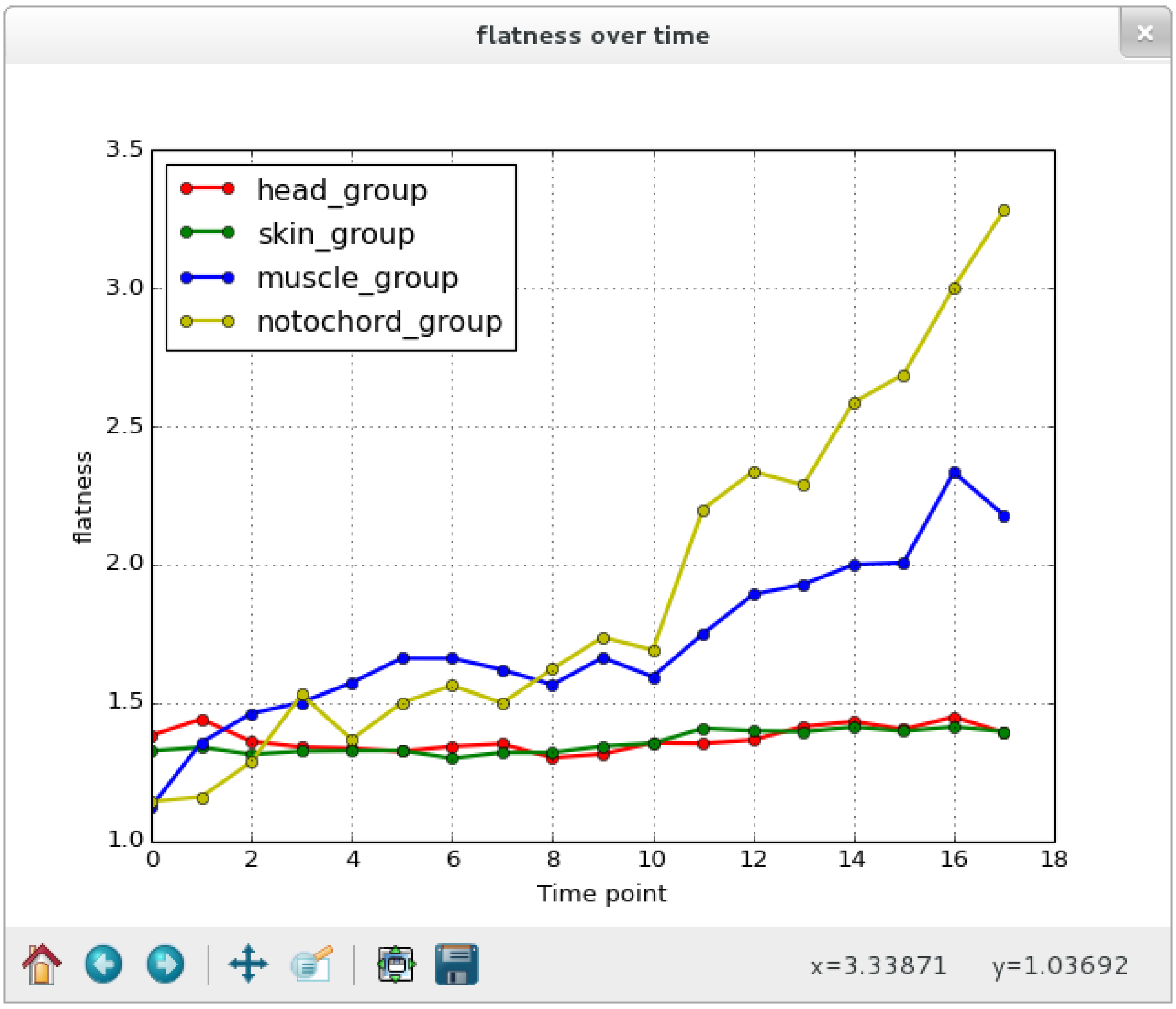

Cylindricity over time in each region of interest. Low values mean more cylindrical.

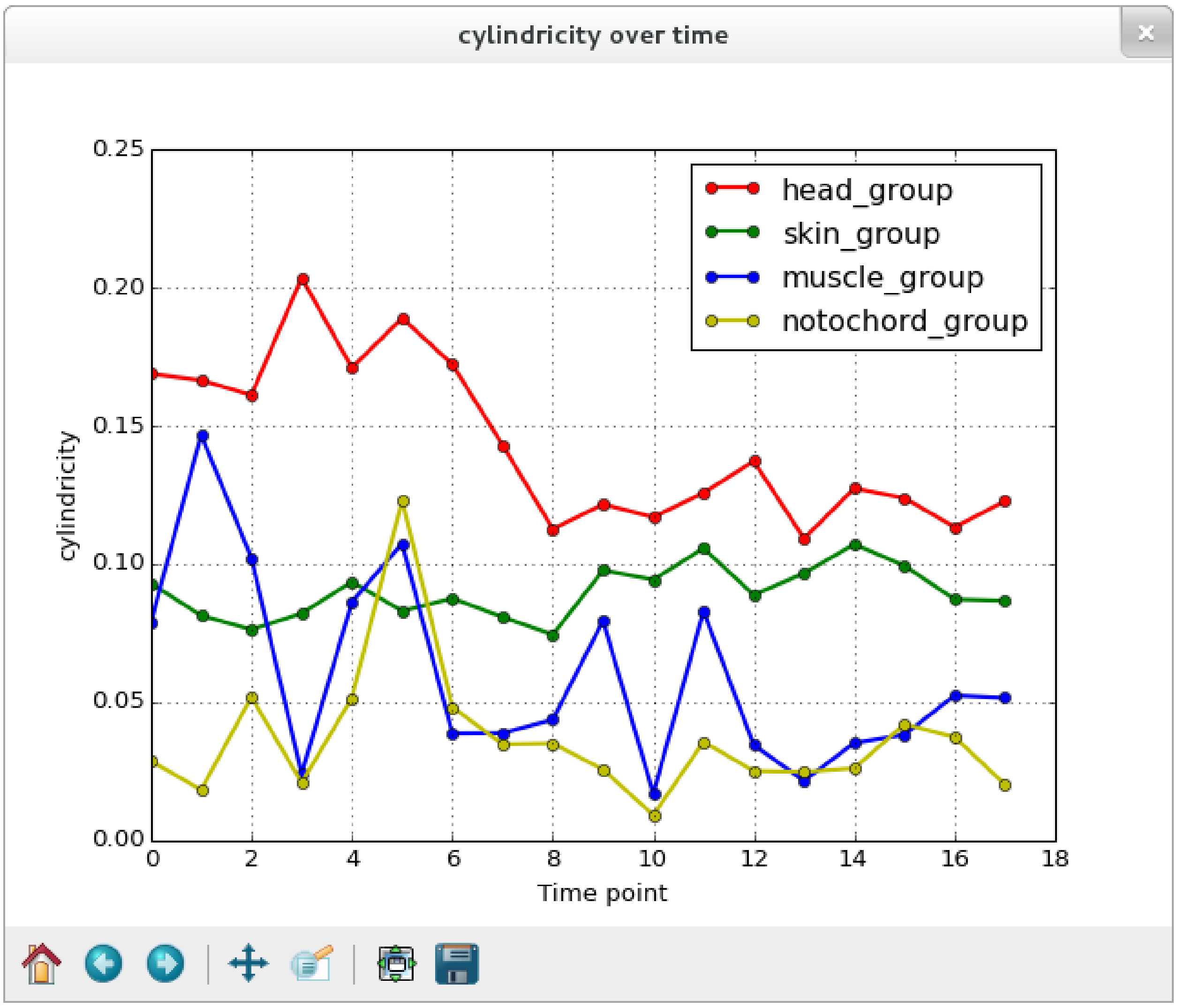

Convexity over time in each region of interest. Low values means more convex.

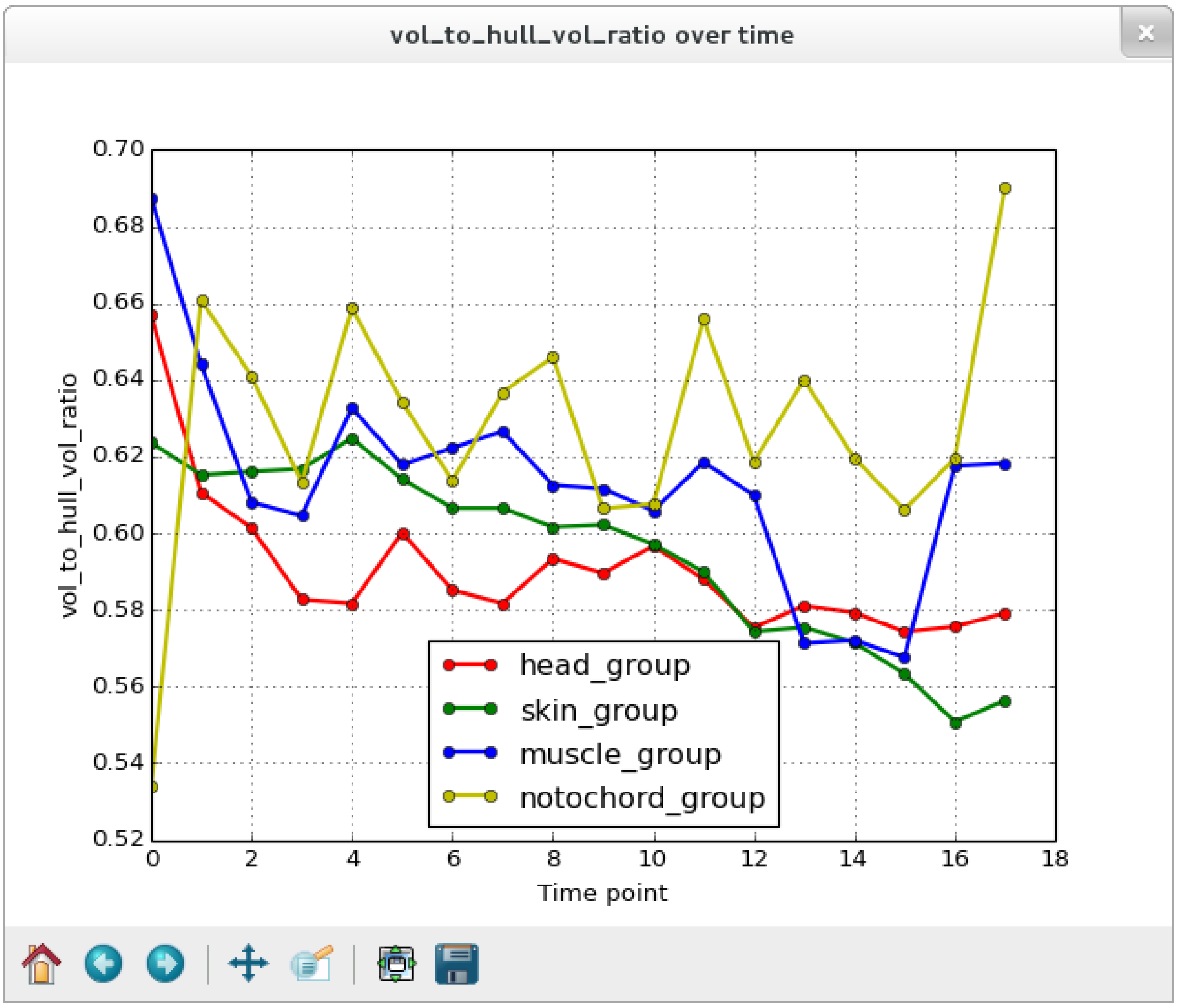

Surface area over time in each region of interest.

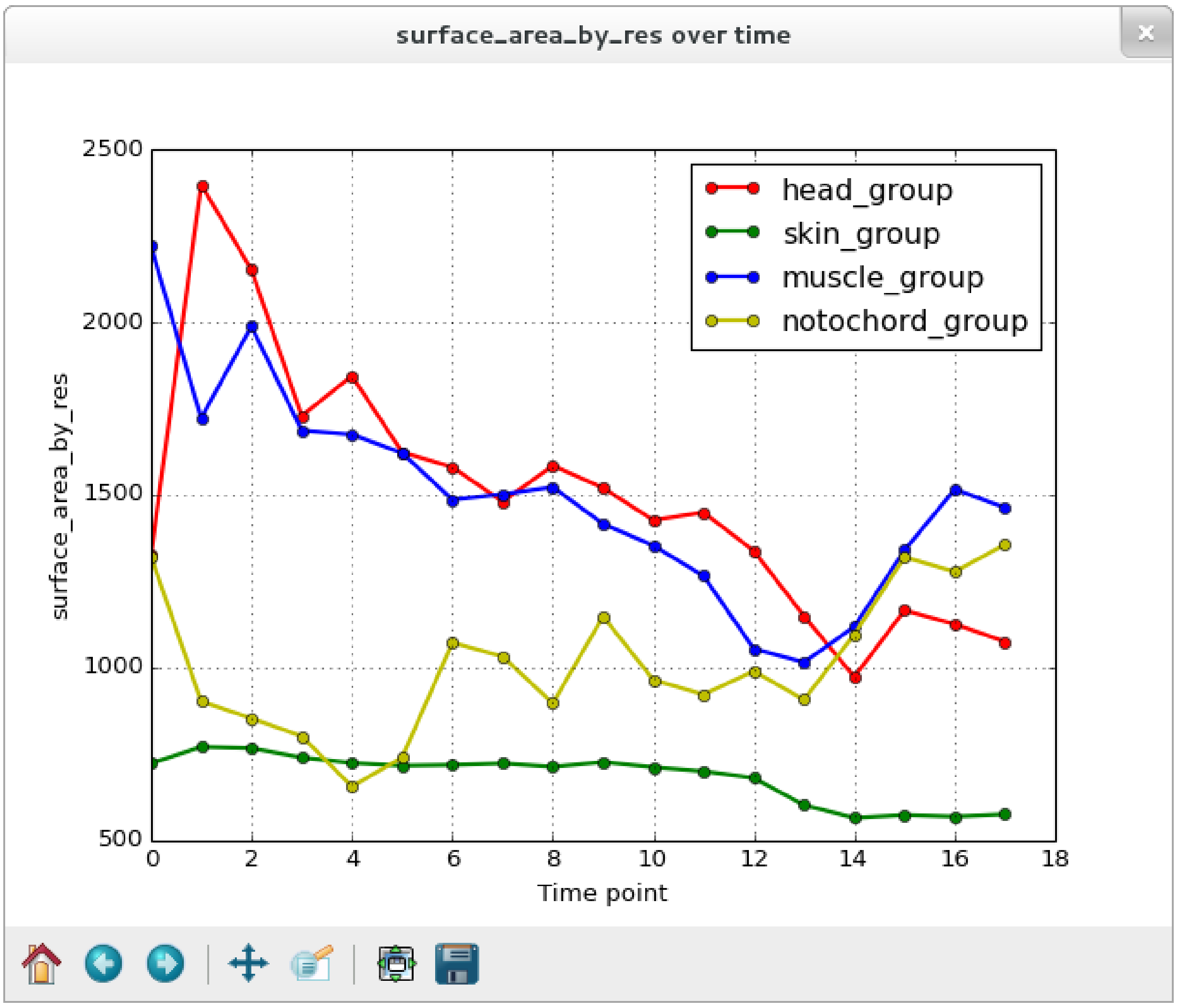

Global histograms over time. Each histogram plot is color coded by time stamp.

Volume histograms over time. (Blue = Early time points, Green = Late time points)

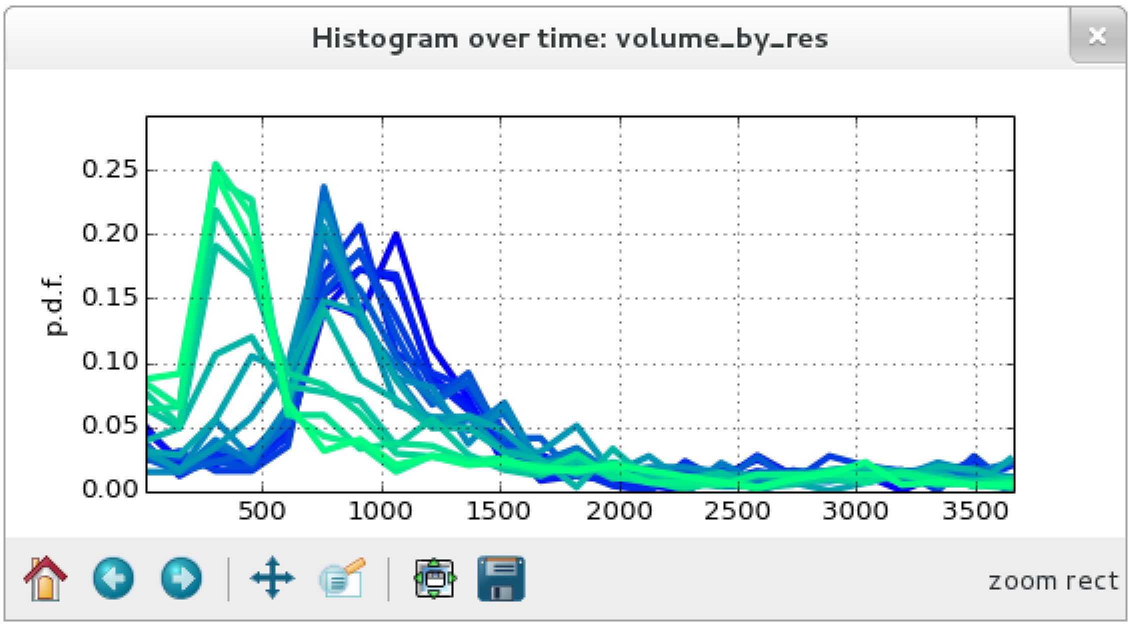

Surface area histograms over time. (Blue = Early time points, Green = Late time points)

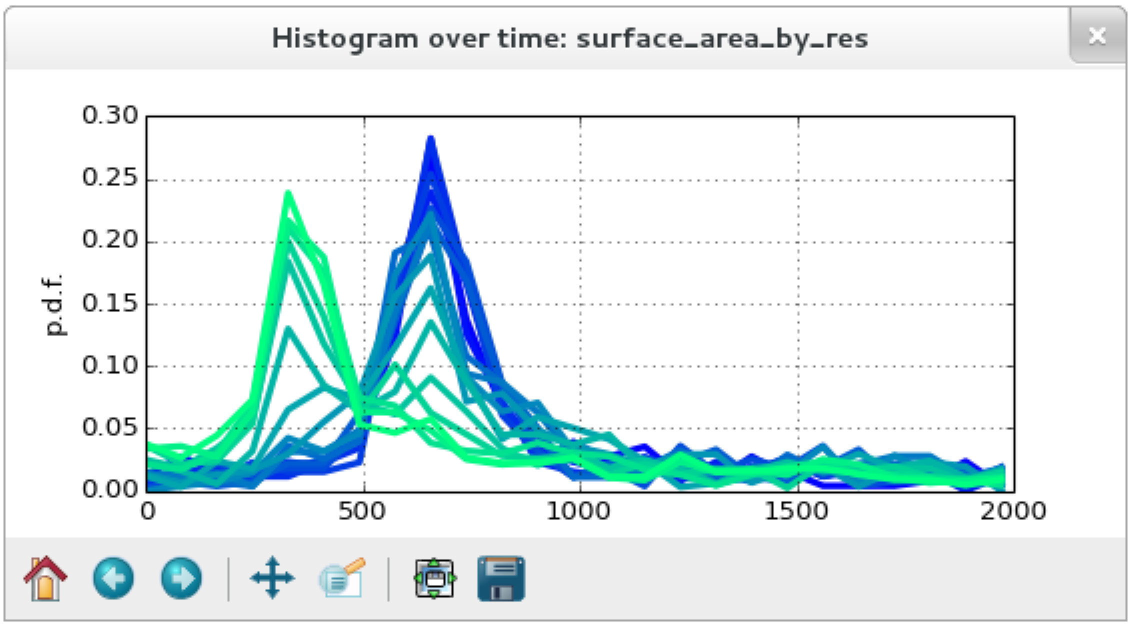

Flatness histograms over time. High values indicate flatter cells. (Blue = Early time points, Green = Late time points)

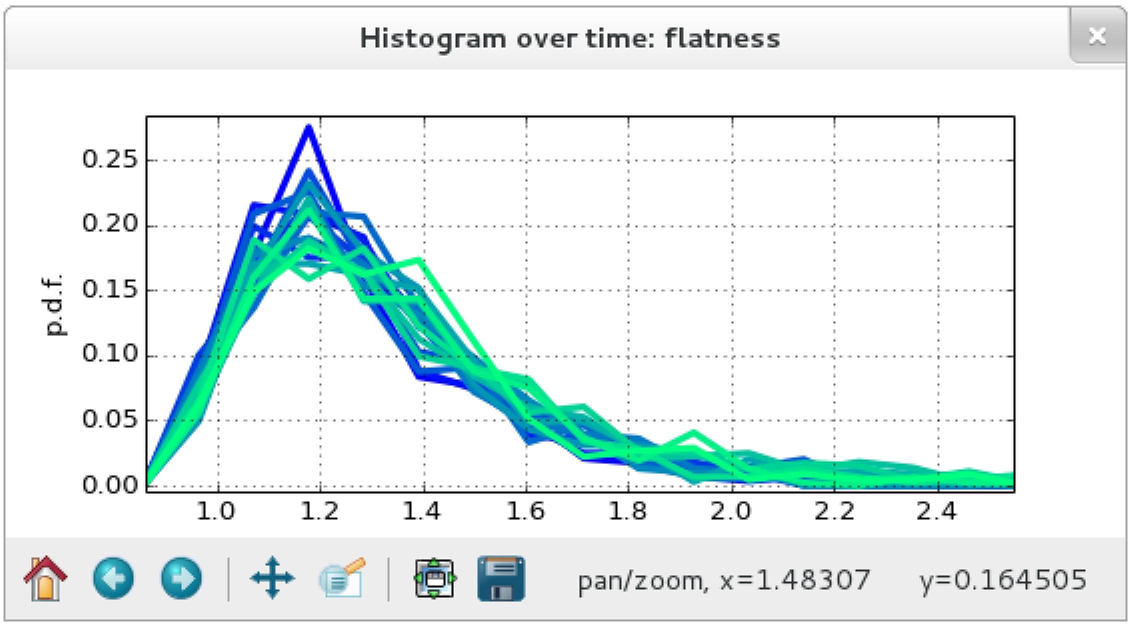

Elongation histograms over time. High values indicate more elongated cells. (Blue = Early time points, Green = Late time points)

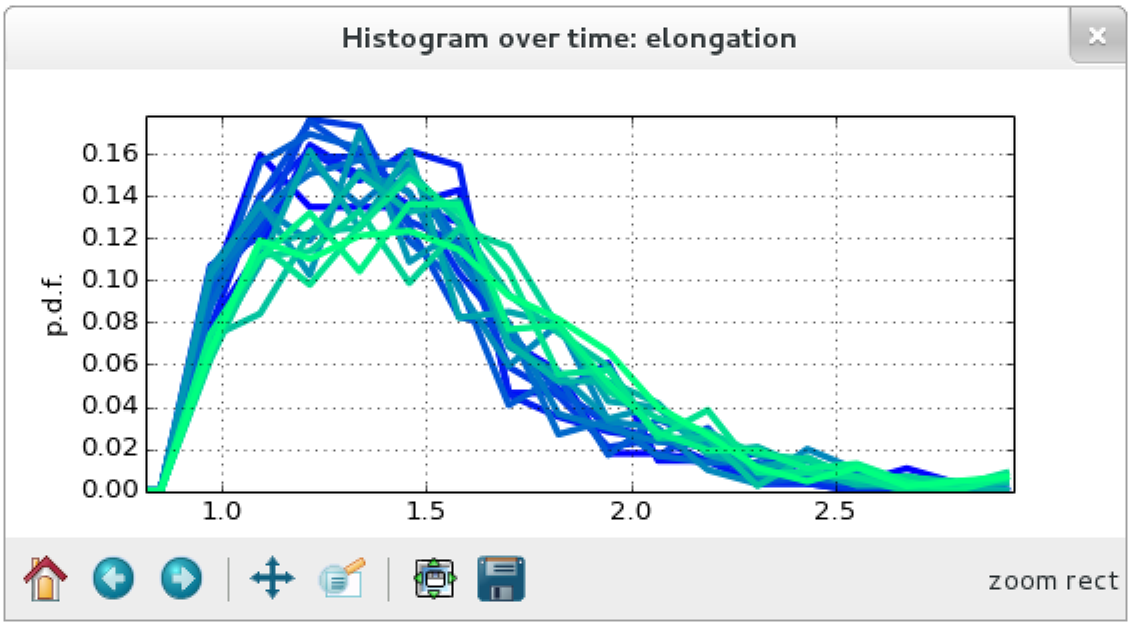

Sphericity histograms over time. Low values indicate more spherical cells. (Blue = Early time points, Green = Late time points)

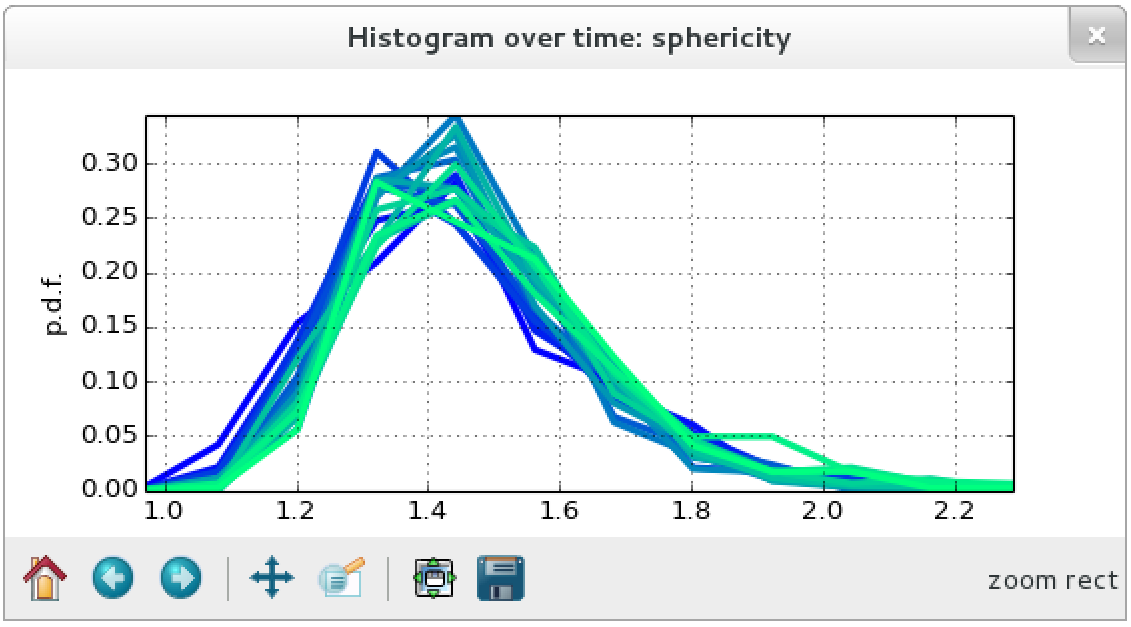

Ascidian-192 Analysis

Global histograms over time. Each histogram plot is color coded by time stamp.

Blue = Early time points
Green = Late time points

Volume histograms over time. (Blue = Early time points, Green = Late time points)

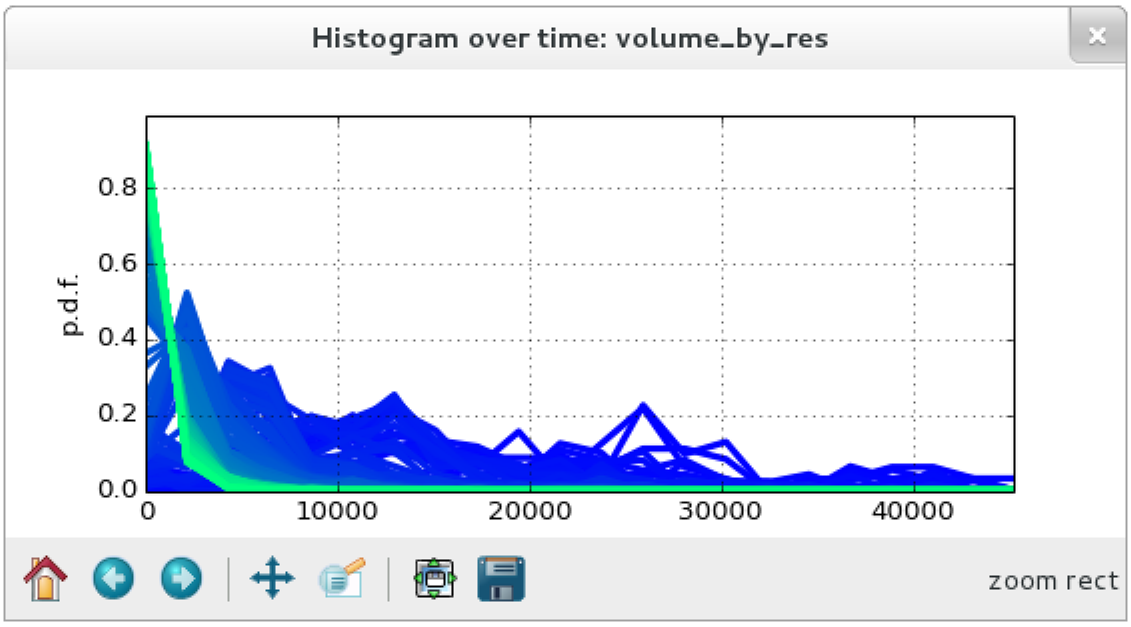

Surface area histograms over time. (Blue = Early time points, Green = Late time points)

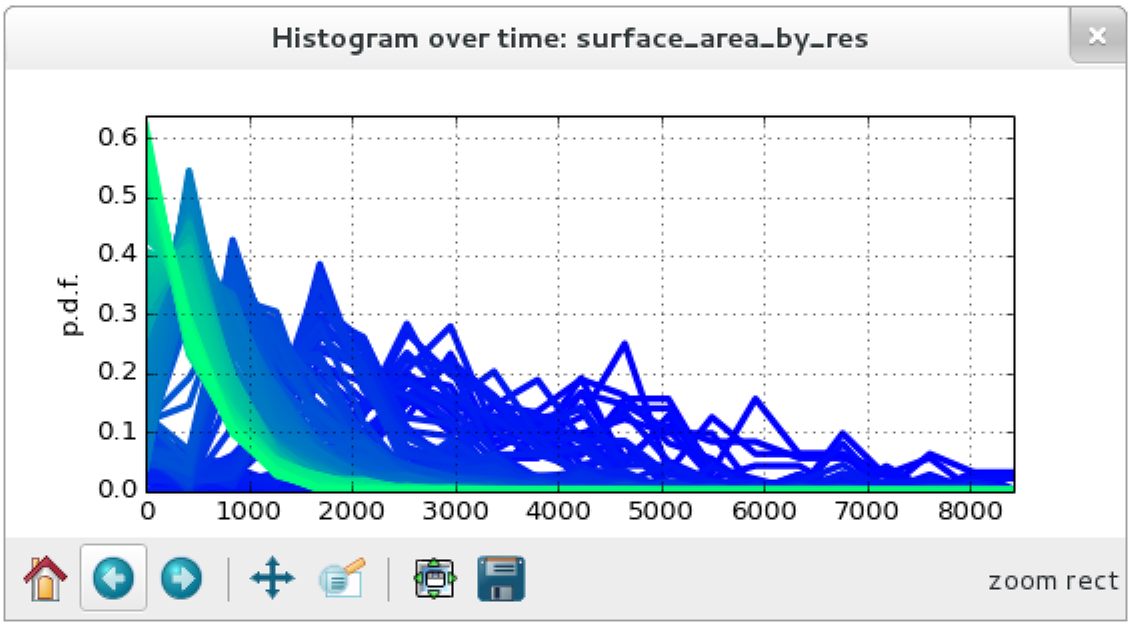

Flatness histograms over time. High values indicate flatter cells. (Blue = Early time points, Green = Late time points)

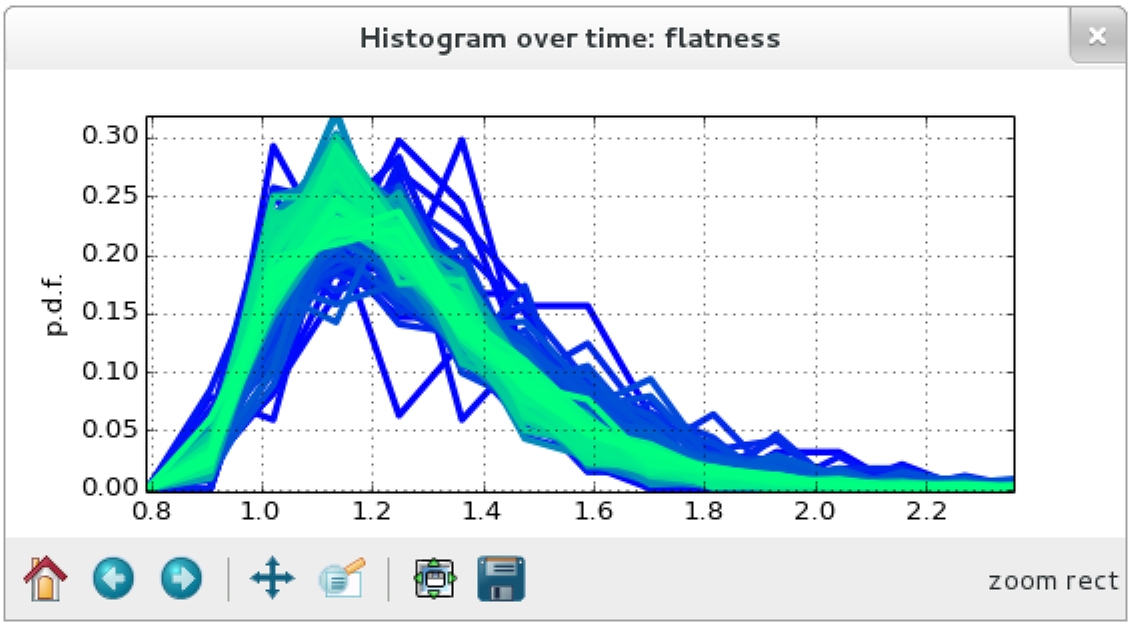

Elongation histograms over time. High values indicate elongated cells. (Blue = Early time points, Green = Late time points)

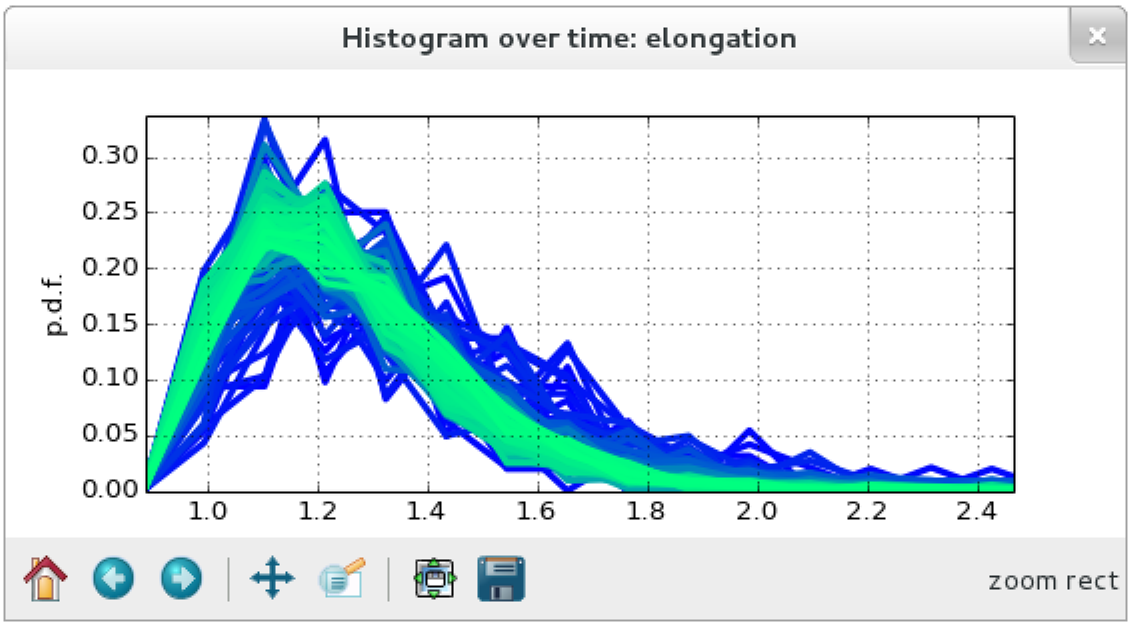

Sphericity histograms over time. Low values indicate spherical cells. (Blue = Early time points, Green = Late time points)

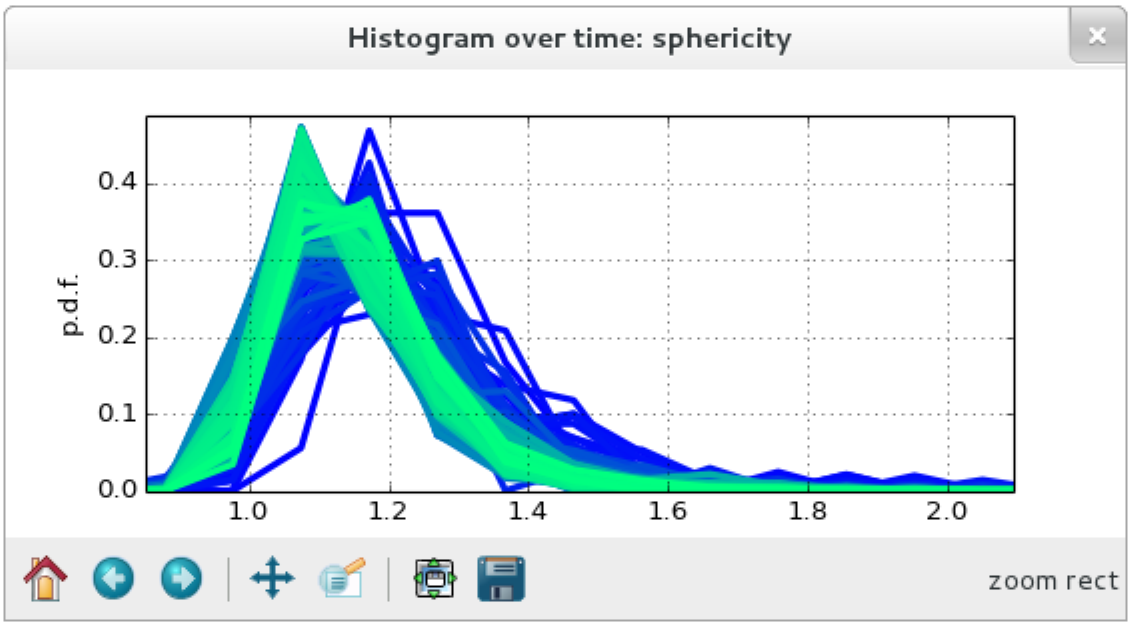

Supplement: Additional file 2 — Additional analysis. Additional plots and measurements obtained on Ascidian-18 and Ascidian-192 datasets. (PDF 4850 kb) [file 12859_2016_927_MOESM2_ESM.pdf]
