# Supplementary figures and images for: CellECT: cell evolution capturing tool
Source: BMC Bioinformatics. 2016 Feb 17;17:88. doi: 10.1186/s12859-016-0927-7 (PMC4756481; doi:10.1186/s12859-016-0927-7)

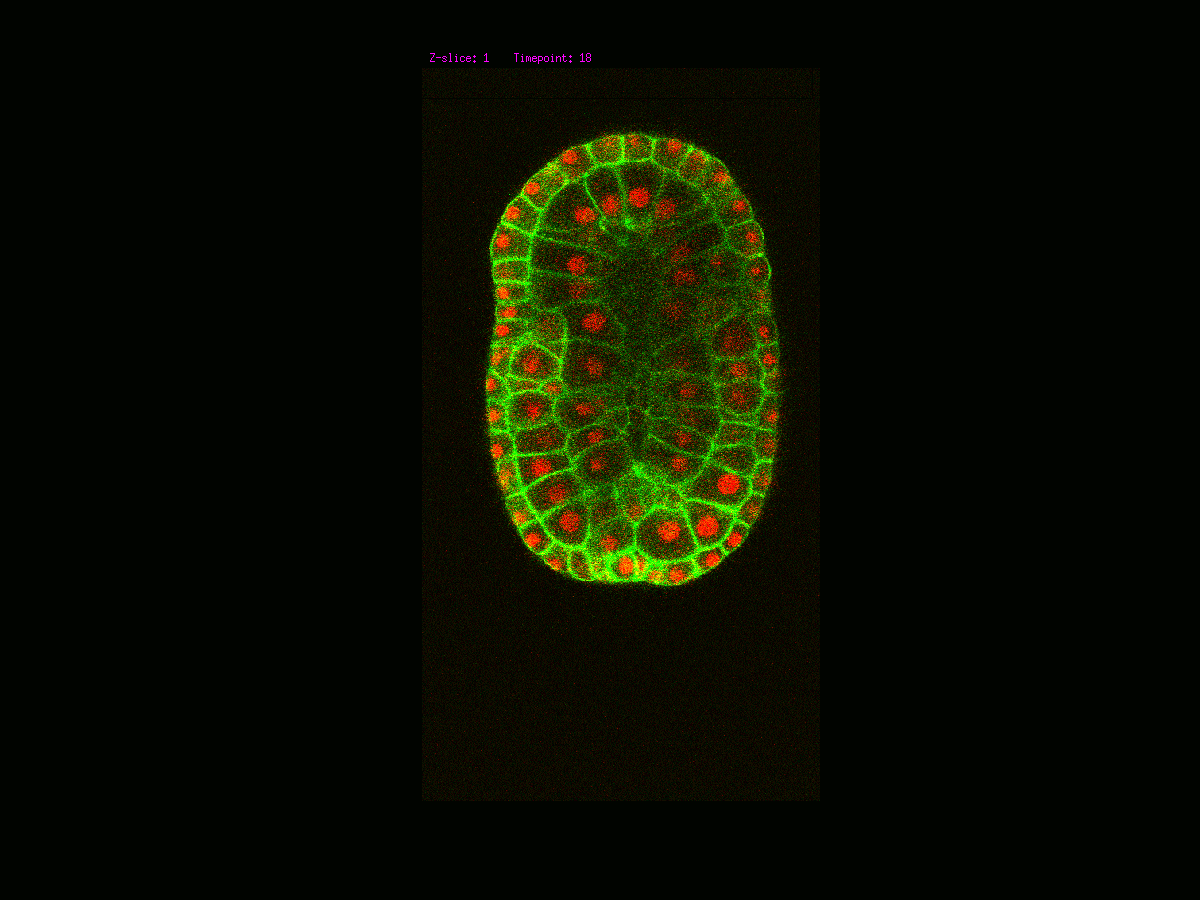

Supplement: Additional file 3 — Ascidian-18 middle slice first to last time point. Animation showing the middle slice of the Ascidian-18 dataset from the first to the last time point. (GIF 4640 kb) [file 12859_2016_927_MOESM3_ESM.gif]

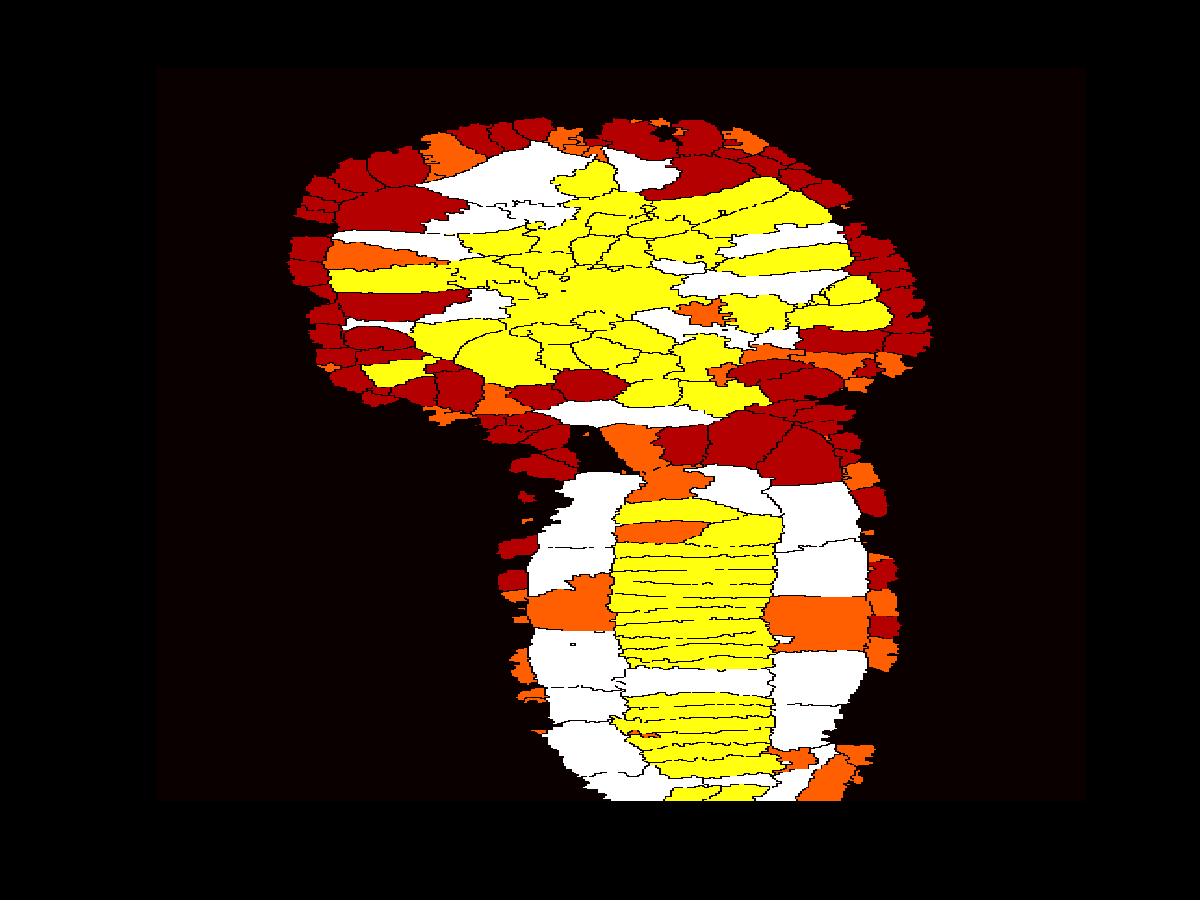

Supplement: Additional file 9 — Cells clustered by features. Animation showing cells clustered by features in the last time point of the Ascidian-18 dataset. (GIF 400 kb) [file 12859_2016_927_MOESM9_ESM.gif]
